# High-pressure pump–probe experiments reveal the mechanism of excited-state proton-coupled electron transfer and a shift from stepwise to concerted pathways

**DOI:** 10.1038/s41557-025-01772-5

**Published:** 2025-03-20

**Authors:** Daniel Langford, Robin Rohr, Stefan Bauroth, Achim Zahl, Alicja Franke, Ivana Ivanović-Burmazović, Dirk M. Guldi

**Affiliations:** 1 FAU Profile Center Solar, Department of Chemistry and Pharmacy and Interdisciplinary Center for Molecular Materials (ICMM), Friedrich-Alexander-Universität Erlangen-Nürnberg, Erlangen, Germany; 2 Department of Chemistry and Pharmacy, Friedrich-Alexander-Universität Erlangen-Nürnberg, Erlangen, Germany; 3 Department of Chemistry, Ludwig-Maximilian-Universität München, Munich, Germany

**Keywords:** Electron transfer, Inorganic chemistry, Reaction kinetics and dynamics, Excited states

## Abstract

Chemical energy conversion and storage in natural and artificial systems rely on proton-coupled electron transfer (PCET) processes. Concerted proton-electron transfer (CPET) can provide kinetic advantages over stepwise processes (electron transfer (ET)/proton transfer (PT) or PT/ET), so understanding how to distinguish and modulate these processes is important for their associated applications. Here, we examined PCET from the excited state of a ruthenium complex under high pressures. At lower buffer or quencher concentrations, a stepwise PT/ET mechanism was observed. With increasing pressure, PT slowed and ET sped up, indicating a merging of the two steps. In contrast, CPET at higher concentrations of buffer or quencher showed no pressure dependence of the reaction rate. This is because the simultaneous transfer of electrons and protons circumvents changes in charges and, consequently, in solvent electrostriction during the transition state. Our findings demonstrate that pressure can serve as a tool to monitor charge changes along PCET pathways, aiding in the identification of its mechanisms.

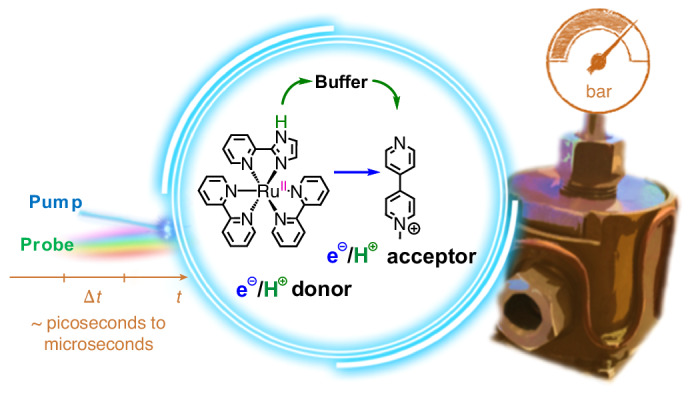

## Abstract (web summary)

Chemical energy conversion and storage rely on the selective movement of protons and electrons, thus understanding these processes is important for applications. Now experiments at elevated pressures are shown to identify excited-state proton-coupled electron transfer mechanisms and to facilitate merging proton transfer with subsequent electron transfer steps towards a concerted pathway.

## Main

Proton-coupled electron transfer (PCET) is nature’s way and means to mediate electric charge carriers^1^. It plays a critical role in a wide array of biochemical redox processes, including respiration complex I^2,3^, cell signalling domains^4^ and DNA biosynthesis for converting nucleotides to 2′-deoxynucleotide^5^. Inspired by nature’s sophisticated light-harvesting techniques, recent research has focussed on photon-to-energy conversion, with an emphasis on chemical fuels^6–8^. Since then, various model systems that mimic the energy conversion and electron transfer (ET) processes in photosynthesis have been developed^8^. These were designed to understand the basic principles of the aforementioned processes^9,10^, including PCET^11–17^, and, ultimately, to integrate them into practical devices^18^. For example, Pannwitz and Wenger reported on electron donor–sensitizer–electron acceptor conjugates based on a modified [Ru(bpy)_3_]^2+^ (where bpy is 2,2′-bipyridine) photosensitizer that mimic the reductions and oxidations seen in photosystem II^19^.

**Fig. 1:**
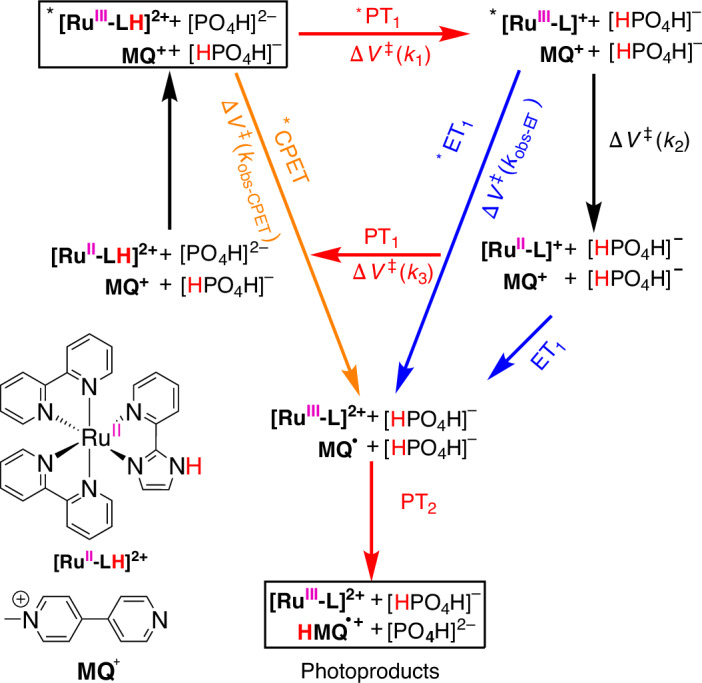
The extended ‘cube’ scheme representing the studied model reaction. The system involves PCET from photoexcited [Ru(bpy)_2_(pyimH)]^2+^ (*[Ru-LH]^**2+**^) to monoquat (**MQ**^**+**^) as the electron acceptor (*ET_1_) and either a buffer or water acting as proton acceptor (*PT_1_)/proton donor (PT_1_ and PT_2_), along with parallel intramolecular quenching (corresponding to *k*_2_). Vertical black arrows depict photoexcitation and deexcitation (that is, intramolecular quenching) processes. Blue arrows, pointing towards the reader, indicate the ET processes to **MQ**^**+**^, with *ET_1_ kinetics determined in this work and ET_1_ thermodynamics taken from ref. ^21^. Red arrows indicate the PT process with the aqueous phosphate buffer. Top: the excited-state chemistry, including PT from the photoexcited complex to the buffer or solvent components (*PT_1_, horizontal red arrow). Middle: PT from the buffer or solvent components to the ground-state complex (PT_1_, horizontal/red arrow). Bottom: PT to the reduced monoquat (PT_2_, vertical red arrow). The diagonal orange arrow indicates the *CPET process, that is, a concerted, bidirectional shift of protons and electrons to the buffer and **MQ**^**+**^, respectively.

Mechanistic PCET studies are often limited to spectral features arising from ET, with proton transfer (PT) frequently remaining undetectable owing to the lack of its visible spectroscopic characteristics. A notable exception is the Haga-type [Ru(bpy)_2_pyimH]^2+^ (**[Ru-LH]**^**2+**^; **LH** = pyimH = 2-(2′-pyridyl)imidazole) complex^20^. In their initial work, Pannwitz and Wenger probed the ET and PT mechanism of **[Ru-LH]**^**2+**^ using nanosecond (ns)-transient absorption spectroscopy (TAS)^21^. Their findings confirmed that **[Ru-LH]**^**2+**^ acts as a photoexcited donor of both protons and electrons in the presence of *N*-methyl-4,4′-bipyridinium (monoquat, **MQ**^**+**^), which serves as a final acceptor for both (Fig. 1)^21^. Either the buffer or the solvent served as proton mediator. The authors demonstrated that photoexcitation of **[Ru-LH]**^**2+**^ substantially decreases the bond dissociation free energy of the imidazole N–H bond, rendering the complex a potent formal hydrogen atom donor^21^. However, a definitive mechanistic assignment—whether it is a concerted (CPET), ET/PT or PT/ET mechanism—was not provided in that work^21^. Addressing this open question regarding the different PCET mechanisms is crucial for controlling and optimizing PCET kinetics. This understanding is essential for advancing catalysis and technologies related to chemical energy conversion and storage, which motivates the present study. Elucidating PCET mechanisms is still a challenging task and is mainly based on interpretations of thermodynamic data, temperature dependence and kinetic isotope effects^22–24^.

Effects of high pressure on the rate and equilibrium of chemical reactions are reflected in the activation volume Δ*V*^‡^ and the reaction volume Δ*V*°, respectively. Both of them are highly sensitive to charge changes or charge distributions^25–28^. This phenomenon is primarily due to electrostriction of solvent in the second coordination sphere and beyond. Two immediate consequences are the release of solvent from the solvation shell to the bulk upon charge reduction and the movement of solvent into the solvation shell upon charge increase^29^. Importantly, in a CPET, changes in charge distribution are absent when the transition state is formed. In contrast, in stepwise ET/PT or PT/ET, charge changes are inevitably. Thus, high-pressure studies, as a means to reflect charge changes along the reaction coordinate, are a powerful tool to distinguish between different types of PCET.

High-pressure methodologies are scarcely used in mechanistic PCET studies^30–34^. In the case of binuclear Ru^II^ complexes, it was possible to differentiate between PCET, pure metal-centred ET or pure ligand-centred ET^31^. Recently, Hammarström et al. applied pressure-dependent optical TAS to study PCET reactions between tungsten hydride complexes and various Ru^III^ or Fe^III^ oxidants^34^. Their study demonstrated the ability to switch between concerted and two stepwise PCET mechanisms predictably^34^. However, the impact of pressure on PCET reactions originating from excited states was not addressed in that work and is explored in the present study.

**Extended Data Fig. 1:**
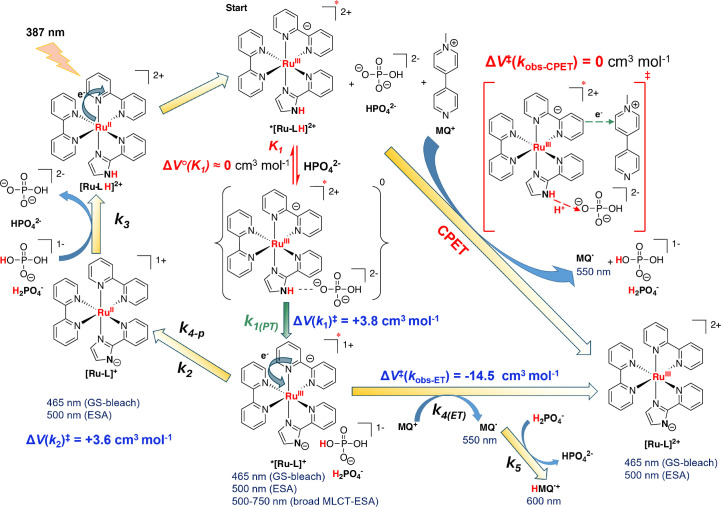
Mechanistic scheme depicting structural formulas of reaction intermediates, with their oxidation and protonation states. Overall mechanistic scheme and overview of experimentally obtained activation and reaction volumes, respectively, for each elementary reaction step, enabling mechanistic assignments under applied experimental conditions (k5 corresponds to PT2 step in Fig. 1).

At the heart of the current work are high-pressure kinetic studies aimed at deciphering reaction mechanisms relevant to solar-to-chemical energy conversion processes, with a particular focus on excited-state PCET. We opted for a PCET evolving from an exited state of the photosensitized *[Ru-LH]^**2+**^ complex, with **MQ**^**+**^ serving as the electron acceptor and either a buffer or water acting as proton acceptor/donor (Fig. 1; for a detailed assignment of the oxidation and protonation state of the reaction intermediates, see Extended Data Fig. 1). At the forefront of this work were pressure-dependent femtosecond (fs)- and nanosecond (ns)-TAS. We started with photoexcited *[Ru-LH]^**2+**^ and progressed via deprotonated *[Ru-L]^**+**^, either towards intermolecular quenching with **MQ**^**+**^ to form **[Ru-L]**^**2+**^ (*ET_1_ in Fig. 1) or towards an intramolecular quenching pathway via **[Ru-L]**^**+**^, ultimately recovering the **[Ru-LH]**^**2+**^ ground state (Fig. 1). The pre-equilibria with buffer components were also studied (vide infra). Pressure responses of underlying reaction steps were quantified by their respective Δ*V*^‡^ or Δ*V*° values. In a time-resolved manner, we monitored the mechanistic changeover from a stepwise to a concerted PCET at high concentrations of **MQ**^**+**^ and buffers. The pressure insensitivity observed for the latter (that is, Δ*V*^‡^ ≈ 0 cm^3^ mol^−1^ for CPET) stands out from the pressure behaviour of all other steps. We also probed whether pressure can modulate a PCET mechanism. Our results demonstrate the potential of using kinetic analyses under different pressures not only to infer charge changes en route to the transition state but also to deduce the character of underlying PCET mechanisms. The most far-reaching aspect of our work is the validation of the pressure paradigm in the context of excited-state reactions, which is relevant for optimizing the photoinduced chemical processes that underpin solar energy conversion. Tuning photoinduced PCET reactions is essential for improving the efficiency of solar energy technologies, such as photocatalysis and solar fuel production, where proper ET and PT is vital^35^.

## Results and discussion

The model reaction depicted in Fig. 1 between the Ru(II) complex in its excited state, *[Ru-LH]^**2+**^, and **MQ**^**+**^ was investigated in a 1:1 (v/v) water/acetonitrile mixture, buffered at pH 6.7 with either phosphate or piperazine-*N*,*N*′-bis-[2-ethanesulfonic acid] (PIPES) (for synthesis, characterization and further experimental description see the Methods). Pressure^-dependent time-resolved fs- and ns-TAS measurements were carried out using a home-made pressure cell for optical measurements suitable for hydrostatic pressure up to 120 MPa that was designed for the purposes of this work (Methods and Supplementary Fig. 1.1).

Before conducting pressure-dependent kinetic studies on the decay of *[Ru-LH]^**2+**^, we examined the contribution of the phosphate buffer to the overall quenching. We started with steady-state emission experiments at a constant pH of 6.7 and different buffer concentrations (Supplementary Fig. 2.1). We observed the emergence of a broad feature between 550 and 800 nm, characterized by a distinct split that suggests the overlapping emissions from the protonated *[Ru-LH]^**2+**^ (at approximately 625 nm), and deprotonated *[Ru-L]^**+**^ (at approximately 675 nm) excited states^21^. The deprotonation in the excited state to yield *[Ru-L]^**+**^ aligns with previous findings regarding the increased acidity of the long-lived triplet metal-to-ligand charge transfer (^3^MLCT) excited state^21^. Given the p*K*_a_* of 5.3 ± 0.6 and p*K*_a_° of 8.1 ± 0.1^21^, at a pH of 6.7, around 96% of the **[Ru-LH]**^**2+**^ is in its protonated form prior to photoexcitation (Supplementary Fig. 2.2a), while photoexcitation results in 96% deprotonation to form *[Ru-L]^**+**^. This shift in photo-acidity supports the hypothesis that the long-lived emissive states are predominantly bipy-^3^MLCT rather than pyimH-^3^MLCT, with a [Ru^III^(bpy)_2_^•─^pyimH]^2+^ character. If 2-(2′-pyridyl)imidazole were to undergo reduction upon excitation, one would expect its excited-state p*K*_a_* to be higher rather than lower compared with the ground-state p*K*_a_° (refs. ^21,36^). Addition of different buffer concentrations to constant **MQ**^**+**^ and **[Ru-LH]**^**2+**^ concentrations induced an emission quenching (Supplementary Fig. 2.1). This quenching confirms that not only the e^−^/H^+^-accepting **MQ**^**+**^ but also the buffer components are involved in the excited-state deactivation, indicating the operation of a PCET mechanism with the participation of buffer molecules as proton donors or acceptors.

To shed light on the PCET mechanism (Fig. 1), we monitored the kinetics of *[Ru-LH]^**2+**^ decay by using TAS measurements in the absence and in the presence of various concentrations of **MQ**^**+**^. Hereby, the **[Ru-LH]**^**2+**^ concentration was kept constant at 0.2 mM using different concentrations of the two buffers, phosphate or PIPES, at pH 6.7 and in the pressure range from 5 to 120 MPa (see Supplementary Sections 3 and 4 for a detailed experimental description).

### Decay kinetics of *[Ru-LH]^2+^ without electron-accepting MQ^+^

To distinguish between intramolecular quenching and intermolecular *ET_1_ to **MQ**^**+**^ (Fig. 1), we initially examined the pressure behaviour of the reaction in the absence of **MQ**^**+**^ at pH 6.7.

**Fig. 2:**
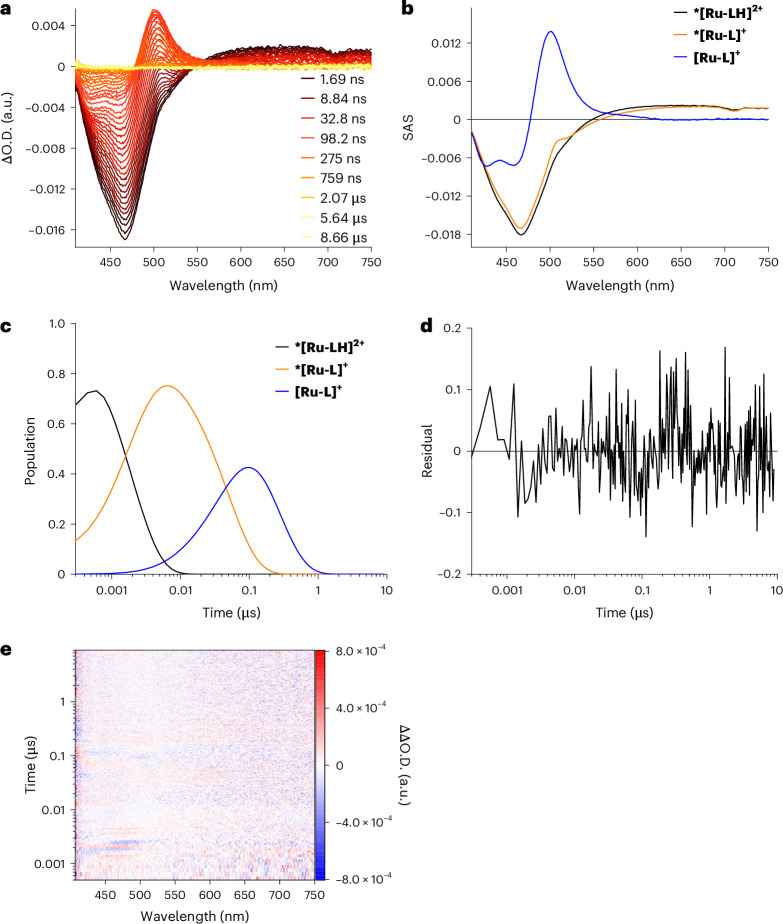
ns-TAS of *[Ru-LH]^2+^ without **MQ**^**+**^ as electron acceptor. **a**, The ns-TAS spectrum recorded for 0.2 mM [**Ru-LH]**^**2+**^ after pulsed laser excitation at 387 nm in a water/acetonitrile solution buffered at pH 6.7 with 50 mM phosphate buffer at 25 °C and 5 MPa pressure (ΔO.D., differential optical density). **b**, The SAS obtained for **a** from three-exponential target analysis with *[Ru-LH]^**2+**^ as the first species (black), *[Ru-L]^**+**^ as the second species (orange) and **[Ru-L]**^**+**^ as the third species (blue). The scaling parameters of the individual reaction steps were adjusted to ensure that the intensity of the SAS at *λ* = 420 nm remained constant. **c**, The corresponding time population profiles from the target analysis. **d**,**e**, The residuals (**d**) and 2D differential residual map (**e**) of the ns-TAS spectra shown in **a** after three-exponential fitting with target analysis. Source data

In the absence of any **MQ**^**+**^, fs-TAS experiments with **[Ru-LH]**^**2+**^ following 387 nm photoexcitation gave rise to excited-state absorptions (ESAs) in the 550–750 nm range and ground-state bleaching at around 465 nm (Supplementary Fig. 3.1a). These ESAs remained persistent throughout the fs-TAS time range and were observable as the initial state in the ns-TAS experiments (Fig. 2a), represented by the black species-associated spectra (SAS) in Fig. 2b. Similar features were noted upon 387-nm photoexcitation of Ru(bpy)_3_ in a water/acetonitrile solution^37^, which confirms the formation of an *[Ru-LH]^**2+**^ excited state that is best described as ^3^MLCT, with an electron delocalized on the bpy ligand (bpy^•─^) rather than on the pyimH ligand.

By monitoring the *[Ru-LH]^**2+**^ relaxation with ns-TAS, we observed that the decay of the ESA above 550 nm is linked to the concomitant formation of a prominent 500-nm ESA (Fig. 2a). This spectral characteristic indicates the generation of the deprotonated **[Ru-L]**^**+**^ ground state. Independent confirmation comes from the difference in the ground-state UV–vis spectra between the deprotonated and protonated complex, which also exhibits the same 500-nm feature (Supplementary Fig. 2.2b).

**Fig. 3:**
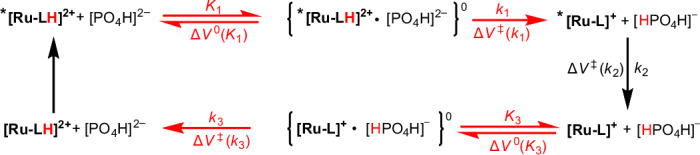
The ‘square’ scheme demonstrating the elementary reaction steps, with the corresponding reaction and activation volumes, in the overall three-step deactivation mechanism of *[Ru-LH]^**2+**^ in the absence of electron accepting **MQ**^**+**^. Vertical black arrows denote photoexcitation and deexcitation processes. Horizontal red arrows in the upper part describe the equilibria/reactions involved in the deprotonation of the photoexcited complex. This is reflected by *PT_1_ in Fig. 1. Horizontal red arrows in the lower part describe the equilibria/reactions involved in the protonation of the ground-state complex. This is reflected by PT_1_ in Fig. 1. All rate and equilibrium constants along with the activation and reaction volumes are summarized in Extended Data Fig. 2 (for a detailed kinetic analysis, see Supplementary Section 3.3).

Considering a direct, one-step conversion from *[Ru-LH]^**2+**^ to the deprotonated **[Ru-L]**^**+**^, followed by the re-protonation of **[Ru-L]**^**+**^ to afford the starting **[Ru-LH]**^**2+**^, we initially applied biexponential target analysis. However, this approach resulted in a rather poor fit with significant residual traces (Supplementary Fig. 3.1). In contrast, a three-species target analysis, consistent with the three steps depicted in the overall reaction scheme in Fig. 3 and the corresponding intermediates (Fig. 2b; black, orange and blue SAS), yielded a good fit (Fig. 2d). These findings suggest the transient formation of the deprotonated excited state *[Ru-L]^**+**^ at pH 6.7, characterized by an isosbestic point around 530 nm (Fig. 2b, orange SAS). Such a mechanism is to be expected, since at pH 6.7 we start from the protonated ground state (p*K*_a_° = 8.1), which upon excitation inevitably undergoes deprotonation, as indicated by its p*K*_a_* of 5.3 (ref. ^21^). Quenching studies across pH levels confirmed single-step quenching under acidic (pH 3.0; Supplementary Fig. 3.2) and basic conditions (pH 9.7; Supplementary Fig. 3.3). However, at pH 6.7, a three-step kinetics model was needed, supporting a *[Ru-LH]^**2+**^ → *[Ru-L]^**+**^ → **[Ru-L]**^**+**^ → **[Ru-LH]**^**2+**^ quenching scheme (Fig. 3 and Supplementary Fig. 3.4d). Similar *[Ru-L]^**+**^ lifetimes across further experiments validated the presence of the same intermediate (for a detailed discussion, see Supplementary Section 3.2).

The re-protonation of **[Ru-L]**^**+**^ at pH 6.7 to finally form **[Ru-LH]**^**2+**^ is evident from the simultaneous disappearance of the prominent 500-nm ESA and the 465-nm ground-state bleaching (Fig. 2a). The overall quenching scheme, involving three consecutive reactions with buffer components as proton donors or acceptors, and the respective related rate constants *k*_1_, *k*_2_ and *k*_3_, is presented in Fig. 3.

Importantly, the observed deprotonation of *[Ru-LH]^**2+**^ to yield *[Ru-L]^**+**^ (see the steps related to *k*_1_ in Fig. 3) is crucial for assigning the PCET mechanism under different experimental conditions (vide infra). This allows us to quantify the pressure effects on pure PT and the subsequent intramolecular quenching (*k*_2_ in Fig. 3). In addition, it helps distinguish this intramolecular pathway from (1) the stepwise PCET quenching pathway that involves intermolecular ET to **MQ**^**+**^ and (2) CPET, which occurs under high **MQ**^**+**^ concentrations (vide infra).

**Fig. 4:**
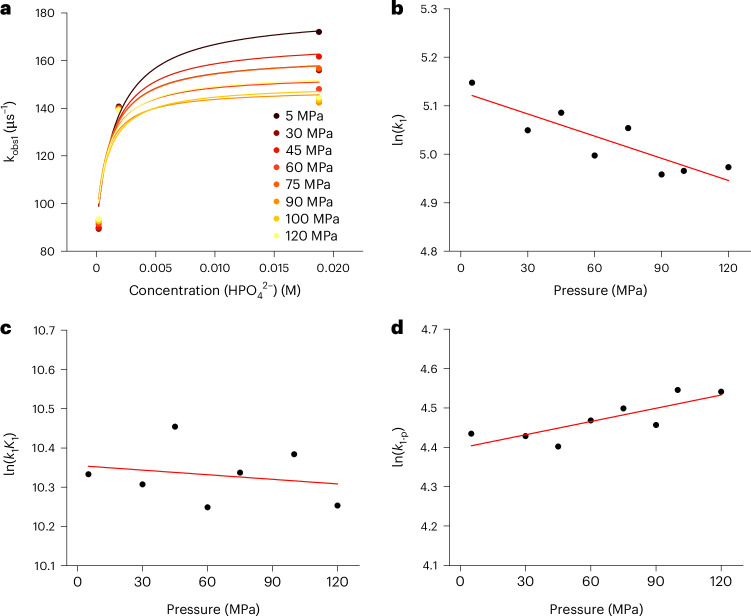
Kinetics of the deprotonation of *[Ru-LH]^2+^ to afford *[Ru-L]^+^ as a function of buffer concentration and pressure. **a**, The dependence of *k*_obs1_ on the concentration of HPO_4_^2−^ as proton-accepting buffer (whose concentration was calculated on the basis of the overall buffer concentration, the pH and its pressure dependence; see Supplementary Section 1.3) and the applied pressure (5–120 MPa) measured at pH 6.7 and T = 298 K. **b**, The pressure dependences of ln(*k*_1_) used to determine Δ*V*^‡^(*k*_1_). **c**, The pressure dependences of ln(*k*_1_*K*_1_) used to determine Δ*V*^0^(*K*_1_). **d**, The pressure dependences of ln(*k*_1-p_) used to determine Δ*V*^‡^(*k*_1-p_). For the values of the activation/reaction volumes of the respective reaction steps, see Extended Data Fig. 2. The parameters for the linear fits depicted in **b**–**d** are summarized in Supplementary Table 3.1. Source data

To quantify the kinetics of each reaction step and determine the activation or reaction volumes for the respective steps, the corresponding apparent rate constants (*k*_obs1_, *k*_obs2_ and *k*_obs3_) were investigated as functions of the phosphate buffer concentration in the pressure range from 5 to 120 MPa (for details, see Supplementary Sections 3.3 and 3.4). The observed rate constants for all three steps showed dependence on the buffer concentration, revealing saturation kinetics (Fig. 4a and Supplementary Figs. 3.7a and 3.8a). Such kinetic behaviour implies an involvement of pre-equilibria (*K*), in which precursor complexes between the Ru species and the corresponding buffer component are formed (see equilibria *K*_1_ and *K*_3_ in Fig. 3). The significant intercepts in the buffer concentration dependence of *k*_obs1_, *k*_obs2_ and *k*_obs3_ indicate that parallel reactions (*k*_1-p_, *k*_2-p_ or *k*_3-p_) occur at each step of the decay kinetics, producing the same products irrespective of the buffer’s presence, involving water molecules as proton acceptors/donors.

The second reaction step is an intramolecular process and is only indirectly dependent on the buffer concentration. This is because the reacting *[Ru-L]^**+**^ is produced in the preceding step involving the phosphate-dependent equilibrium K_1_. This equilibrium affects the concentration of *[Ru-L]^**+**^ and consequently its rate constant *k*_obs2_, which varies with the phosphate concentration until saturation is reached (Supplementary Fig. 3.7a). Therefore, only the pressure dependence of *k*_obs2_ at the highest buffer concentration (saturated conditions) and for the parallel process (*k*_2-p_), both unaffected by buffer concentration, is quantified (Supplementary Fig. 3.7b,c).

**Extended Data Fig. 2:**
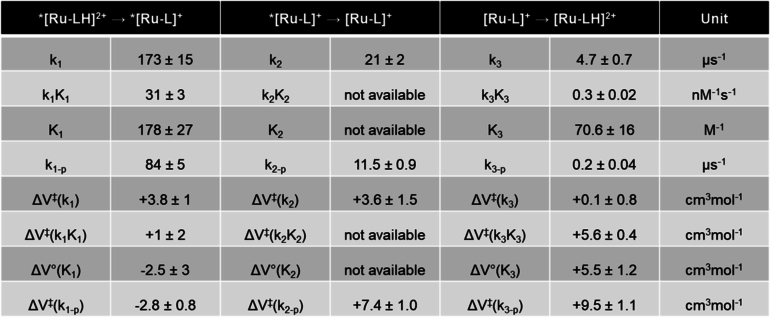
Kinetic and thermodynamic data on the decay of the excited state in the absence of an electron acceptor. Summary of the rate constants, equilibrium constants with phosphate buffer components and corresponding activation and reaction volumes, ΔV^‡^ and ΔV° respectively, for the *[Ru LH]^2+^ decay in the absence of electron accepting MQ^+^.

An important finding is that *K*_1_ is pressure independent within experimental error limits, with ΔV^0^(*K*_1_) ≈ 0 cm^3^ mol^−1^. This results from two opposing effects: the formation of the precursor {*[Ru-LH]^**2+**^•HPO_4_^2−^} is associated with a volume decrease, while the neutralization of the charges induces a volume increase due to reduced electrostriction. These effects cancel each other out, resulting in negligible volume changes. Similar effects occur with *K*_3_, where {**[Ru-L]**^**+**^•H_2_PO_4_^−^} is less compact owing to the lower ionic charges, leading to a positive reaction volume of Δ*V*^0^(*K*_3_) = 5.5 ± 1.2 cm^3^ mol^−1^ (Extended Data Fig. 2). From the pressure independence of *K*_1_ and the positive value of Δ*V*^‡^(*k*_1_), it is inferred that pressure decelerates the overall PT from *[Ru-LH]^**2+**^ to the buffer. The activation volume Δ*V*^‡^(*k*_1_) for PT within {*[Ru-LH]^**2+**^•HPO_4_^2−^} is similar to the Δ*V*^‡^(*k*_2_) observed for subsequent ground-state recovery. Both steps involve charge redistribution en route to the transition state, whether through proton movement within {*[Ru-LH]^**2+**^•HPO_4_^2−^} or through charge redistribution within the discrete complex *[Ru-L]^**+**^ (Fig. 3, *k*_2_ path). Likewise, charge redistribution within {**[Ru-L]**^**+**^•H_2_PO_4_^−^} also impacts the generation of a transition state along the *k*_3_ step. However, here, proton movement increases charges, inducing increased electrostriction and lowering Δ*V*^‡^(*k*_3_) compared with Δ*V*^‡^(*k*_1_) and Δ*V*^‡^(*k*_2_). To probe whether the *[Ru-LH]^**2+**^ decay is buffer specific, similar TAS measurements were performed at pH 6.7 with a PIPES buffer (Supplementary Section 3.5). The observation of the same reaction sequence as with phosphate buffer confirmed a general, non-specific involvement of the buffer in PT from or to the imidazole fragments.

### Decay kinetics of *[Ru-LH]^2+^ with electron-accepting MQ^+^

Under our reaction conditions, namely at pH 6.7, **MQ**^**+**^ is fully deprotonated as its p*K*_a_ (HMQ^2+^/MQ^+^) is 3.0. It becomes, however, protonated after reduction to form **HMQ**^**•+**^ owing to its p*K*_a_ (HMQ^•+^/MQ^•^) of 10.4 (ref. ^21^). The reaction free energy for the PCET reaction from *[Ru-LH]^**2+**^ to **MQ**^**+**^ to afford **[Ru-L]**^**2+**^ and **HMQ**^**•+**^, calculated from the formal N–H bond dissociation free energies (−10 ± 6 kcal mol^−1^), implies a thermodynamically favourable reaction^21^. In aqueous buffered solutions, the reaction between two positively charged ions *[Ru-LH]^**2+**^ and **MQ**^**+**^ proceeds rather through a bidirectional mechanism, where photoinduced ET to **MQ**^**+**^ is coupled to PT from pyimH to the buffer^21^. Pannwitz and Wenger found that both concerted and stepwise mechanisms are compatible with their experimental data^21^.

In the present study, we focussed on the high-pressure approach to distinguish between these two different mechanisms. Activation volumes for CPET are expected to be close to zero, as the simultaneous transfer of protons and electrons does not result in notable charge changes at the transition state. In stark contrast, a rate-limiting PT is likely to be associated with a substantially large Δ*V*^‡^, similar to that observed in pure protolytic reactions. If ET controls the reaction mechanism, a different change in the activation volume is expected. Specifically, an increase in positive charge from +1 in *[Ru-L]^**+**^ to +2 in **[Ru-L]**^**2+**^, resulting from ET to **MQ**^**+**^, should result in a negative activation volume^38,39^. This is because an increase in charge at the transition state correlates with heightened electrostriction of the surrounding solvent molecules, making the solvation sphere more compact. Conversely, the charge neutralization from **MQ**^**+**^ to **MQ**^**•**^ upon accepting an electron should not substantially impact Δ*V*^‡^ or Δ*V*^0^, as changes in delocalized charges generally do not result in large volume changes^31,38,39^. In short, Δ*V*^‡^ resulting from ET is expected to be influenced by the formation of **[Ru-L]**^**2+**^ rather than **MQ**^**•**^. We aimed to probe this paradigm through our high-pressure experiments.

**Fig. 5:**
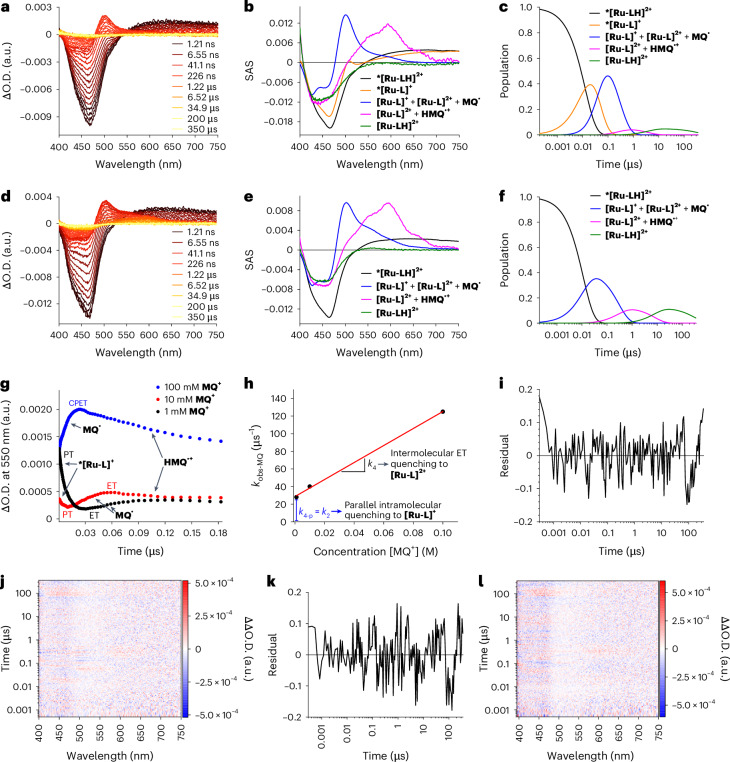
Decay of 0.2 mM *[Ru-LH]^**2**+^ (photoexcitation at 387 nm in water/acetonitrile with 50 mM phosphate buffer at pH 6.7, 25 °C and 5 MPa) at variable **MQ**^**+**^ concentrations and kinetics of ET. **a**, ns-TAS spectra with 10 mM **MQ**^**+**^. **b**, SAS obtained for the reaction in **a** from the target analysis for *[Ru-LH]^**2+**^ (black), *[Ru-L]^**+**^ (orange), **[Ru-L]**^**+**^ + **[Ru-L]**^**2+**^ + **MQ**^**•**^ (blue), **[Ru-L]**^**2+**^ + **HMQ**^**•+**^ (pink) and residual **[Ru-LH]**^**2+**^ ground-state bleach (green). The green SAS in **b** and **e** was necessary for target analysis owing to the non-quantitative recovery of the **[Ru-LH]**^**2+**^ ground state at the end of the time scale. **c**, The time-resolved population profile of the corresponding SAS, obtained from target analysis of **a**. The green profile in **c** and **f**, though required in target analysis, reflects only incomplete **[Ru-LH]**^**2+**^ ground-state recovery and provides no kinetic information. **d**, The ns-TAS spectra with 100 mM **MQ**^**+**^. **e**, The SAS obtained for the reaction in **d** from the target analysis of *[Ru-LH]^**2+**^ (black), **[Ru-L]**^**+**^ + **[Ru-L]**^**2+**^ + **MQ**^**•**^ (blue), **[Ru-L]**^**2+**^ + **HMQ**^**•+**^ (pink) and residual **[Ru-LH]**^**2+**^ ground-state bleach as a result of incomplete thermal reverse PCET between **[Ru-L]**^**2+**^ and **HMQ**^**•**^^**+**^ (green). **f**, The time-resolved population profile of the corresponding SAS, obtained from target analysis of **d**. **g**, The kinetic traces recorded at 550 nm for the reaction in **a** and **d** in the presence of various concentrations of **MQ**^**+**^. **h**, A linear plot of *k*_obs-MQ_ versus the concentration of **MQ**^**+**^ to determine the second-order rate constant for ET. The parameters for the linear fit are summarized in Supplementary Table 3.1. **i**,**j**, The residual trace (**i**) and two-dimensional (2D) differential residual map (**j**) of the ns-TAS spectra shown in **a** after fitting by target analysis. **k**,**l**, The residual trace (**k**) and 2D differential residual map (**l**) of the ns-TAS spectra shown in **d** after fitting by target analysis. The scaling parameters of the individual reaction steps were adjusted to ensure that the intensity of the SAS at *λ* = 420 nm remained constant. The jump in the residual traces at 80 µs noted in **i** and **k** is a result of an instrument artefact caused by light scattering. Source data

The mechanism of the *[Ru-LH]^**2+**^ decay was monitored at different **MQ**^**+**^ concentrations within a pressure range of 5–120 MPa in either 50 mM phosphate or PIPES buffer, at pH 6.7 and room temperature (RT). In all TAS experiments, the first SAS obtained corresponded to *[Ru-LH]^**2+**^ (Fig. 5, black SAS). Target analyses of the TAS data (Fig. 5a) required a five-step fitting model for the *[Ru-LH]^**2+**^ decay to initial **[Ru-LH]**^**2+**^ at low **MQ**^**+**^ concentration (10 mM, Fig. 5b, c; for 1 mM **MQ**^**+**^, see Supplementary Fig. 4.1). In contrast, a four-step fitting model was sufficient for high **MQ**^**+**^ concentrations (100 mM; Fig. 5d–f and Supplementary Fig. 4.2).

At low **MQ**^**+**^ concentrations, *[Ru-LH]^**2+**^ first decays into a species characterized by a superposition of ESA in the 550–750 nm range and at 500 nm (orange SAS in Fig. 5b and Supplementary Fig. 4.1). This species corresponds to the deprotonated excited state *[Ru-L]^**+**^, which is also characterized as such by independent experiments in the absence of **MQ**^**+**^ and by varying pH (orange SAS in Fig. 2b and Supplementary Fig. 3.4d; Supplementary Sections 3.1 and 3.2). In the subsequent step, *[Ru-L]^**+**^ undergoes ET to **MQ**^**+**^, yielding **[Ru-L]**^**2+**^ (characterized by ESA at 500 nm) and **MQ**^**•**^ (characterized by ESA at 550 nm), as evident from the blue SAS in Fig. 5b and Supplementary Fig. 4.1. These findings support a stepwise PT-ET mechanism at low **MQ**^**+**^ concentrations.

At high **MQ**^**+**^ concentrations, however, *[Ru-LH]^**2+**^ decays directly, in a single step, to the oxidized and deprotonated **[Ru-L]**^**2+**^ along with the generation of **MQ**^**•**^. This is evidenced by the mono-exponential rise of the 500- and 550-nm ESAs accompanied by a quantitative depopulation of the ESA in the 550–750 nm range (Fig. 5e, blue SAS), bypassing the intermediate formation of the deprotonated excited state *[Ru-L]^**+**^. This suggests simultaneous transfer of the electron to **MQ**^**+**^ and the proton to the buffer, according to a bidirectional CPET mechanism. To calculate the blue SAS under conditions of low and high **MQ**^**+**^ concentration, we employed a kinetic model that accounts for the contributions of three distinct species: the deprotonated **[Ru-L]**^**+**^, the deprotonated and oxidized **[Ru-L]**^**2+**^ and the reduced **MQ**^**•**^. The first species arises from competing intramolecular quenching, while the latter two are products of the intermolecular ET reaction and were assumed to be formed in equimolar amounts.

All subsequent species were identical in the experiments at both low and high **MQ**^**+**^ concentration. Specifically, **MQ**^**•**^ is consecutively protonated by the buffer to yield **HMQ**^**•+**^, with a characteristic 600-nm feature observed in the third SAS in the high concentration regime (Fig. 5e, pink SAS) or in the fourth SAS in the low concentration regime (Fig. 5b, pink SAS). The final step in both regimes is the recovery of the protonated ground state **[Ru-LH]**^**2+**^_._

Notably, the corresponding SAS (for example, the blue or pink SAS shown in Fig. 5 or Supplementary Section 4), calculated from data obtained under different reaction conditions (for example, changes in buffer and/or **MQ**^**+**^ concentrations), differ in both shape and intensity. This variation arises because, while the SAS represent the superposition of the same species, their relative contributions (concentrations) depend on the reaction conditions and the differing optical properties of the individual species. For instance, the blue SAS, which combines the contributions of **[Ru-L]**^**+**^, **[Ru-L]**^**2+**^ and **MQ**^**•**^, is dominated by the optical characteristics of **[Ru-L]**^**+**^ under high buffer and low **MQ**^**+**^ concentrations. However, under low buffer and high **MQ**^**+**^ concentrations, the spectral profile is primarily shaped by **[Ru-L]**^**2+**^and **MQ**^**•**^. Similarly, the pink SAS is largely influenced by the 600-nm feature of **HMQ**^**•+**^, which becomes dominant at moderate to high **MQ**^**+**^ concentrations (10 mM and 100 mM) combined with a high buffer concentration (≥5 mM).

Based on the spectro-electrochemical data from the literature, the 500-nm feature observable at both high and low concentrations of **MQ**^**+**^ (Fig. 5a,b,d,e and Supplementary Fig. 4.1) can be ascribed to the oxidized and deprotonated **[Ru-L]**^**2+**^. However, the intensity and the shape of the 500-nm ESA resemble those of **[Ru-L]**^**+**^, the species observed when the reaction is carried out without **MQ**^**+**^ (see the blue SAS in Fig. 2b and Supplementary Fig. 3.4d). This suggests that, besides a bidirectional PCET from *[Ru-LH]^**2+**^, a parallel intramolecular quenching to the deprotonated ground state **[Ru-L]**^**+**^ occurs (Figs. 1 and 3, *k*_2_ path). Thus, some *[Ru-LH]^**2+**^ undergoes PCET quenching (Fig. 1, *PT_1_ followed by *ET_1_), resulting in **[Ru-L]**^**2+**^, while another subset of *[Ru-LH]^**2+**^ relaxes intramolecularly to **[Ru-L]**^**+**^ upon deprotonation (Fig. 1, *k*_2_ path). To confirm the operation of such parallel process, we opted for monitoring the 500- and 550-nm ESA intensity as functions of the phosphate buffer concentration at a constant **MQ**^**+**^ concentration of 100 mM. As depicted in Supplementary Fig. 4.3c, a higher concentration of buffer correlates with a higher ratio of ESA(500 nm)/ESA(550 nm). This indicates a competition reaction involving the buffer molecules, which becomes faster and more efficient with increasing buffer concentrations. Conversely, as shown in Supplementary Fig. 4.3d, under conditions of constant buffer concentration (50 mM) but increasing **MQ**^**+**^ concentration (1, 10 and 100 mM), the ratio ESA(500 nm)/ESA(550 nm) decreases notably, indicating a more efficient PCET reaction under these conditions (Fig. 1, path *ET1 or *CPET). The occurrence of parallel reactions was also observed by Wenger et al.^21^. All of them depend on the relative ratio of buffer and **MQ**^**+**^ concentrations (Supplementary Fig. 4.3).

The operation of different mechanisms for *[Ru-LH]^**2+**^ quenching at low and high **MQ**^**+**^ concentration was also evident from the temporal evolution of the ESA at 550 nm, related to the formation of **MQ**^**•**^ (Fig. 5g). At low **MQ**^**+**^ concentrations (1 and 10 mM; Fig. 5g, black and red curves), PT and ET are well separated. However, at higher concentrations, **MQ**^**•**^ formation is substantially accelerated, while the PT rate, solely dependent on buffer concentration, remains unaffected. Thus, at 100 mM **MQ**^**+**^, the formation of *[Ru-L]^**+**^ becomes unresolvable (Fig. 5e and blue curve in Fig. 5g) owing to the accelerated subsequent reactions. This suggests a merging of stepwise PT and ET into a single CPET process. However, a stepwise mechanism cannot be excluded solely because the intermediate *[Ru-L]^**+**^, formed during PT, is undetectable. A slow PT followed by a fast ET may also explain our observations. To clarify the mechanism, further kinetic studies of **MQ**^**•**^ formation were performed.

**Fig. 6:**
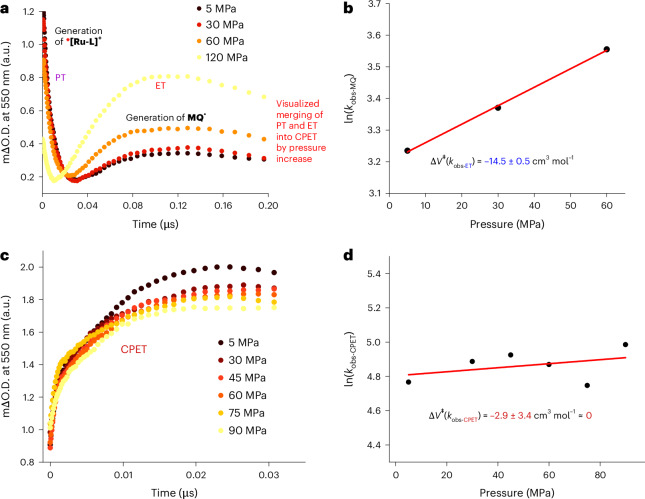
The pressure response of ET, at low **MQ**^**+**^ concentrations, versus CPET, at high MQ^+^ concentrations. **a**, The kinetic traces recorded at 550 nm for **MQ**^**•+**^ formation after pulsed laser excitation at 387 nm of 0.2 mM [Ru(bpy)_2_pyimH]^2+^ in the presence of the lowest **MQ**^**+**^ concentration of 1 mM in water/acetonitrile solution buffered at pH 6.7 (50 mM phosphate buffer) at 25 °C in the pressure range of 5–120 MPa. **b**, The pressure dependence of ln(*k*_obs-ET_) used to determine the activation volume for ET to **MQ**^**+**^, constructed on the basis of the data shown in **a**. **c**, The kinetic traces recorded at 550 nm for CPET after pulsed laser excitation at 387 nm of 0.2 mM [Ru(bpy)_2_pyimH]^2+^ in the presence of the highest **MQ**^**+**^ concentration of 100 mM in water/acetonitrile solution buffered at pH 6.7 (50 mM phosphate buffer) at 25 °C in the pressure range of 5–90 MPa. **d**, The pressure dependence of ln(*k*_obs-CPET_) used to determine the value of the activation volume for CPET, constructed on the basis of the data shown in **c**. The values of *k*_obs_ (*k*_obs-ET_ and *k*_obs-CPET_) obtained from the fit of experimental data to a single exponential function (Extended Data Figs. 3 and 4) as well as the parameters for the linear fits depicted in **b** and **d** are summarized in Extended Data Fig. 5 and Supplementary Table 3.1, respectively. Source data

Initially, the pseudo-first order rate constant for the formation of **MQ**^**•**^ (*k*_obs-MQ_) was studied as a function of the **MQ**^**+**^ concentration in 50 mM phosphate buffer at pH 6.7, RT and ambient pressure. To isolate the changes originating from possible initial generation of *[Ru-L]^**+**^, the *k*_obs-MQ_ for **MQ**^**•**^ formation was determined from the kinetics at 536 nm, which is the isosbestic point for *[Ru-L]^**+**^. A representative trace with a mono-exponential fit is shown in Supplementary Fig. 4.4. *k*_obs-MQ_ increases linearly with [**MQ**^**+**^] and shows a significant intercept (Fig. 5h). From the linear plot of *k*_obs-MQ_ versus **MQ**^**+**^ concentration, the second-order rate constant for **MQ**^**•**^ formation was determined to be *k*_4_ = (9.6 ± 0.3) × 10^8^ M^−1^ s^−1^. The intercept is *k*_4-p_ = 28.7 ± 1.8 µs^−1^. The *k*_4_ value is approximately four times higher than the PCET quenching constant obtained indirectly by Wenger et al. in Stern–Volmer experiments (*k*_q_ = (2.1 ± 0.4) × 10^8^ M^−1^ s^−1^), where a 5 mM acetate buffer at pH 6.3 was used^21^. A non-zero intercept indicates a parallel, competitive reaction, mediated by the buffer as described above (Fig. 5h). This *k*_4-p_ intercept is close to *k*_2_ = 21 µs^−1^, determined in experiments without **MQ**^**+**^ and conducted at the highest phosphate buffer concentration of 50 mM (see Extended Data Fig. 2 and the saturation value for *k*_obs2_ in Supplementary Fig. 3.7a). These results affirm the nature of the parallel reaction as the quenching of *[Ru-L]^**+**^ to yield **[Ru-L]**^**+**^ (*k*_2_ in Figs. 3 and 6), having identical lifetimes in various experiments, that is, in Britton–Robinson buffer at pH 9.7 and 6.7 without **MQ**^**+**^ as well as in phosphate buffer at pH 6.7 with and without **MQ**^**+**^ (details provided earlier and in Supplementary Section 3.2).

To shed light on the underlying PCET mechanism, the generation of **MQ**^**•**^ was studied as a function of pressure at every concentration of **MQ**^**+**^. Absorbance changes due to either PT or ET are well separated for pressures of up to 60 MPa (Fig. 6a). This allowed us to determine the activation volume for ET to **MQ**^**+**^ (*ET_1_ in Fig. 1), that is, ΔV^‡^(*k*_obs-ET_), as −14.5 ± 0.5 cm^3^ mol^−1^ (Fig. 6b). Considering that Δ*V*^‡^(*k*_obs-ET_) also includes volume changes associated with the parallel reaction with buffer (corresponding to Δ*V*^‡^(*k*_2_) = +3.6 ± 1.5 cm^3^ mol^−1^ in Extended Data Fig. 2 and Fig. 1), an even more negative Δ*V*^‡^ is likely to evolve for the **MQ**^**+**^ reduction. As discussed above, this is in line with what is expectable for Δ*V*^‡^ for ET. Although this volume collapse when approaching the transition state can partly be attributed to the intrinsic volume changes in the Ru complex due to oxidation to Ru^III^, the primary effect is related to increased electrostriction and enhanced solvation. This is caused by the rise in the overall charge of the Ru complex from +1 to +2.

Approaching higher concentrations of **MQ**^**+**^ (Fig. 6c), PT and ET reactions occur simultaneously, indicating a CPET mechanism (Fig. 1, orange *CPET path). Our pressure studies revealed a Δ*V*^‡^(*k*_obs-CPET_) of −2.9 ± 3.4 cm^3^ mol^−1^, which, considering the error limits, is very close to zero (Fig. 6d).

These results imply a transition state in which the deprotonation of *[Ru-LH]^**2+**^ is accompanied by ET from its bpy^•−^ to **MQ**^**+**^. Consequently, no substantial change in the overall charge of the Ru complex is expected when reaching the transition state, resulting in minimal electrostriction effects, as predicted for a CPET mechanism. This concerted bidirectional PCET mechanism, involving separate proton and electron acceptors, can be seen as a trimolecular event (Extended Data Fig. 1, CPET path). This agrees with the experimental observation that high concentrations of both **MQ**^**+**^ and phosphate buffer are required for CPET to occur. As shown in Supplementary Figs. 4.5 and 4.6, at lower phosphate concentrations (0.5 mM and 5 mM), and regardless of a high [**MQ**^**+**^] of 100 mM, the formation of transient *[Ru-L]^**+**^ occurs, indicating a stepwise PT/ET mechanism. Overall, our results validate the use of pressure studies for investigating excited-state PCET, where kinetic measurements under different pressures provide a unique tool for distinguishing between stepwise and concerted processes. (For details on the final steps, the protonation of **MQ**^**•**^ and thermal reverse PCET, see Supplementary Section 4.1.5.)

## Conclusion

In this work, we demonstrated the power of high-pressure kinetic studies in elucidating the mechanisms of PCET processes, particularly in the excited state. By observing the pressure-induced deceleration of PT and acceleration of ET, we showed a trend towards the convergence of the two steps, aligning with the positive activation volume for PT and negative activation volume for ET. Although a complete transition from stepwise PT/ET to concerted PCET was not observed up to 120 MPa, higher pressures may be needed for such a shift. At this point, we wonder whether it is possible to transform a concerted into a stepwise PCET mechanism or if we can change a stepwise PT/ET (or ET/PT) mechanism, going over a CPET mechanism, into a ET/PT (or PT/ET) mechanism by changing the pressure (Extended Data Fig. 6). This would imply the presence of ‘asynchronous’ pressure effects on individual ET and PT processes as parts of a concerted mechanism, which seems inconsistent with the tunnelling theory of PCET that involves electrons and protons tunnelling via the same transition state of CPET^22,34^. Our results demonstrate that applying pressures in the range of 5–90 MPa does not transform CPET into a stepwise mechanism. However, there have been reports of asynchronous concerted mechanisms^40–42^, suggesting that higher pressures may induce a shift from CPET to a stepwise mechanism. Further studies of CPET at elevated pressures, supported by theory, are needed to obtain a definitive answer. We also believe that utilizing pressure in PCET research holds vast potential for exploring other fundamental aspects. For instance, investigating the impact of the proton tunnelling distance on the CPET rate through variable-pressure studies would be an excellent addition to the existing experimental research on the topic^43,44^.

## Methods

### Synthetic methods

All chemicals and solvents were of p.a. grade and were purchased from Sigma-Aldrich or abcr GmbH if not mentioned otherwise. All syntheses were carried out under an inert argon atmosphere using standard Schlenk techniques.

### Synthesis of [Ru(bpy)_2_(pyimH)]^2+^[ClO_4_^−^]_2_

The synthesis of [Ru(bpy)_2_(pyimH)][ClO_4_]_2_ was based on the procedure reported by Haga^20^. *cis*-Ru(bpy)_2_Cl_2_ (75 mg, 0.16 mmol, 1.0 eq) was suspended in a mixture of EtOH and H_2_O (v/v 1:1, 20 mL) in an argon atmosphere. The reaction mixture was refluxed for 30 min and cooled to RT, and 2-(2′-pyridyl)imidazole (pyimH) (26 mg, 0.18 mmol, 1.1 eq) was added. The reaction mixture was kept under reflux conditions for 3 h. The reddish solution was concentrated in vacuo to remove all ethanol, and solid NaClO_4_ (0.52 mmol, 3.4 eq) was added. The resulting voluminous orange–red solid (69 mg) was filtered and recrystallized in MeOH (15 mL). Yield: 80%

^1^H-NMR (400 MHz, CD_3_CN) *δ* = 8.46 (m, 4H, H_arom_), 8.15 (d, 1H, J = 8.0 Hz, H_arom_), 8.02 (m, 5H, H_arom_), 7.80 (m, 4H, H_arom_), 7.60 (d, 1H, J = 4.0 Hz, H_imidazole_), 7.38 (m, 6H, H_arom_), 6.55 (d, 1H, J = 4.0 Hz, H_imidazole_) ppm.

^13^C {^1^H} NMR (101 MHz, CD_3_CN) *δ* = 158.67 (C_26_), 158.52, 158.11, 158.09, 153.06, 153.00, 152.96, 152.90, 152.75, 149.98, 147.77 (C_1_, C_5_, C_6_, C_10_, C_11_, C_15_, C_16_, C_20_, C_21_, C_25_), 138.85, 138.29, 138.22, 138.14, 138.12 (C_3_, C_8_, C_13_, C_18_, C_23_), 129.55, 128.35, 128.33 (C_4_, C_7_, C_14_), 128.24, 127.81, 126.94 (C_17_, C_24_, C_27_), 125.05, 124.89, 124.66, 124.64, 123.15 (C_2_, C_9_, C_12_, C_19_, C_22_), 122.65 (C_28_) ppm.

Elemental analysis calculated for C_28_H_23_N_7_Ru·2ClO_4_ + 2NaClO_4_ (956.40 g mol^−1^): C 33.55%, H 2.31 %, N 9.78 %; found: C 33.28%, H 2.51%, N 9.98%.

Electrospray ionization (ESI)-mass spectrometry (MS) (MeOH): *m*/*z* (%) = 279.55 [M]^2+^ (100), 558.10 [M–H]^+^ (16).

### Synthesis of *N*-methyl-4,4′-bipyridinium hexafluorophosphate

*N*-methyl-4,4′-bipyridinium hexafluorophosphate was synthesized acoording to literature known conditions^45^. The 4,4′-Bipyridine (6.4 mmol, 1 equiv.) was dissolved in acetonitrile, and methyl iodide (6.4 mmol, 1 equiv.) was added dropwise. The solution was stirred for 12 h at RT. After filtration and washing with chloroform and ether, an orange product was obtained. The solid was dissolved and treated with ammonium hexafluorophosphate to isolate the final product. Yield: 65%

^1^H NMR (400 MHz, D_2_O) *δ* = 8.88 (d), 8.74 (d), 8.61 (d), 8.04 (d), 4.40 (s) ppm.

^13^C {^1^H} NMR (101 MHz, D_2_O) δ = 153.50, 151.95, 150.86, 142.12, 124.77, 121.86, 47.22 ppm.

ESI-MS (MeOH): *m*/*z* (%) = 171.08 [M]^+^ (100).

### Spectroscopic methods—general remarks

High-resolution (HR)-ESI-MS was performed on a Bruker Daltonik (Bremen, Germany) maXis plus, equipped with a quadrupole time-of-flight (qToF) detector. Detection was in positive ion mode with a source voltage of 3.8 kV. The flow rates were 180 μl h^−1^. The drying gas (N_2_) used to aid solvent removal was held at 180 °C. The MS was calibrated before every experiment via direct infusion of the Agilent ESI-TOF low concentration tuning mixture.

NMR measurements were performed on a Bruker AVANCE DRX400 WB instrument.

Electrochemical measurements were performed by using an Autolab PGSTAT 101 device (Metrohm). A conventional three-electrode arrangement was employed, consisting of a platinum working electrode (geometric area 0.07 cm^2^; Metrohm), a platinum wire auxiliary electrode (Metrohm) and a Ag/AgCl, LiCl (3 M, in EtOH; Metrohm) reference electrode. All electrochemical measurements were performed in CH_2_Cl_2_ with 0.1 M NBu_4_BF_4_ as supporting electrolyte. All solutions were thoroughly degassed with N_2_ before use, and a stream of N_2_ was maintained throughout the measurement. The solutions were thermostated at 21 °C.

### Spectroscopic methods—TAS

Transient absorption (TA) measurements with the phosphate or PIPES buffered systems were carried out on a HELIOS femtosecond spectrometer and EOS nanosecond spectrometer (Ultrafast Systems). The light source was a Clark MXR CPA 2110 Ti:sapphire amplifier with an output of 150-fs light pulses at 775 nm and a repetition rate of 1,050 Hz. The excitation pulses at 387 nm with an energy of 800 nJ were generated via second harmonic generation. The white light in the visible range (420–760 nm) used for the probe pulse in the femtosecond experiments was generated by focussing the 775-nm fundamental onto a 2-mm sapphire disk. For the nanosecond experiments, a supercontinuum laser source (370–1,600 nm) with a 2,100 Hz repetition rate and a pulse width of approximately 1 ns was used.

TAS measurements with the Britton–Robinson buffered systems were carried out with a HELIOS femtosecond spectrometer and a EOS nanosecond spectrometer (Ultrafast Systems). The light source was an Astrella-F-1K (Coherent Inc.) with a repetition rate of 1 kHz and 5.0 W power output at 800 nm (5 mJ pulse energy and pulse duration of 80 fs). Of the laser output, 1.2 mJ was used to pump a Topas Prime from Light Conversion with a standard NIRUVIS extension module. The white light in the visible range (450–760 nm) used for the probe pulse in the femtosecond experiments was generated by focussing the 800-nm fundamental onto a 2-mm sapphire disk. For the nanosecond experiments, a supercontinuum laser source (370–1,600 nm) with a 2 kHz repetition rate and a pulse width of approximately 1 ns was used.

It is important to clarify that we do not record kinetic traces at different wavelengths but rather time-resolved Vis spectra at different time intervals after the excitation pulse. The kinetic traces we show correspond to the changes in optical density as a function of time. We employ pulsed laser spectroscopy operating at a repetition rate of 1,050 Hz (for phosphate and PIPES buffer) or 1 kHz (for Britton–Robinson buffer). For the femtosecond transient absorption spectra (1 ps to 5 ns), every second pulse is used to measure the Vis spectrum before photoexcitation, alternating with every other pulse used to measure the Vis spectrum after photoexcitation. These alternatingly measured Vis spectra, each measured with a repetition rate of 525 Hz (or 500 Hz, respectively), are automatically combined to the obtained differential absorption spectrum. Each differential spectrum was averaged for 1 s. In total, 250 time steps were recorded and the entire time range was scanned three times. The average of these three scans is used to generate the depicted spectra. For the nanosecond transient absorption spectroscopy in the time range from 1 ns to 360 µs, Vis spectra were recorded at different time intervals relative to the excitation pulse. Here, the white-light pulse comes from an additional white-light supercontinuum laser, which is electronically triggered. The time axis is not linearly scanned as in femtosecond transient absorption spectroscopy. Instead, the time points are randomly selected and recorded. The accumulation of these recorded spectra is directly represented as an averaged product. Typically, with a measurement duration of 20 min per spectrum, each time point is measured about 1,000 times.

### Pressure methods

An in-house-developed pressure setup was used for the pressure-dependent TA experiments. The pressure apparatus used is shown in Supplementary Fig. 1.1. The sample was placed in a cylindrical cuvette with an inner diameter of 4 mm and sealed with a moveable stopper. The cuvette was placed in the pressure cell, which was filled with water as the pressure medium. The pressure of the medium was built up by turning the wheel of the pressure pump and transferred through the coil to the cell.

The pressure apparatus was equipped with the sample, pressurized to a maximum pressure of 1,200 bar and left for equilibration before the first measurement was started. The system was repeatedly checked for sudden drops of pressure and leakage of the joints. The pressure was typically changed in 300–450-bar steps and left for equilibration before the subsequent measurement was started. All systems were measured once in the depressurization direction from 1,200 to 50 bar and subsequently in the pressurization direction from 50 to 1,200 bar to check the reversibility of the pressure effect on the kinetics.

Since the phosphate buffer used for the pressure measurements exhibits a pressure-dependent p*K*_a_ (Δ*V*_a_° = −25.85 ± 0.02 cm^3^ mol^−1^)^46^ at the studied pH, the appropriate changes in pH and the associated changes in the concentration of HPO_4_^2−^/H_2_PO_4_^−^ species on going from 5 to 120 MPa were taken into consideration for the calculations of the corresponding values of activation/reaction volumes.

### Method for global and target analysis

All fs- and ns-TAS spectra were fitted by using the GloTarAn software package^47^. The spectra were deconvoluted by fitting with a linear combination of exponential functions. Each exponential function was represented with a lifetime on the time axis and the corresponding spectrum at the given lifetime on the spectral axis. The lifetimes are represented on population–time plots, and the corresponding spectra are referred to as EAS for global analysis or SAS for target analysis. Global analysis with a sequential evolution model was used for the reference experiments with the Britton–Robinson buffer at pH 3.0 and 9.7. All other measurements were modelled with a competing ground-state deactivation path in a target model. The scaling parameters of the individual reaction steps were adjusted so that the intensity of the SAS at *λ* = 420 nm remained constant, as this position is least influenced by changes in neighbouring spectral bands. No change of the spectral features upon pressure change was observable in our investigated systems. The differences between the recorded spectrum and the linear combination of the convoluted lifetimes and EAS/SAS are depicted as residual traces. Good quality of fit is defined as the lowest number of exponential functions that result in exclusively statistical noise in the residual trace without any observable kinetic traces.

### Kinetic analysis of *k*_obs1_, *k*_obs2_ and *k*_obs3_ as functions of buffer concentration and pressure without MQ^+^

A full description of the method applied is given in the Supplementary Information.

## Online content

Any methods, additional references, Nature Portfolio reporting summaries, source data, extended data, supplementary information, acknowledgements, peer review information; details of author contributions and competing interests; and statements of data and code availability are available at 10.1038/s41557-025-01772-5.

## Supplementary information

Supplementary Information Supplementary Figs. 1.1, 2.1-2.2, 3.1-3.13 and 4.1-4.14, Tables 3.1 and 3.2, Discussion and Kinetic analysis.

## Source data

Source Data Fig. 2 Datasets for ns-TAS spectra, SAS with corresponding time population profiles and residual traces of ns-TAS spectra.

Source Data Fig. 4 Kinetic data (rate constants) as a function of concentration and pressure.

Source Data Fig. 5 Datasets for TAS spectra, time-resolved population profiles with residual traces of ns-TAS spectra, kinetic trace (Abs versus time) and kinetic data (rate constants) as a function of concentration.

Source Data Fig. 6 Datasets for kinetic traces (Abs versus time) and rate constants as a function of pressure.

**Extended Data Fig. 3:**
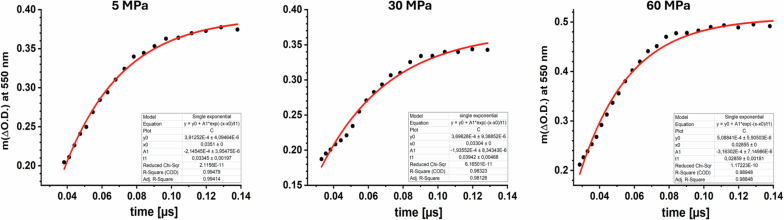
Determination of pseudo-first order rate constants (k_obs-ET_) for the *ET reaction between *[Ru L]^+^ and MQ+. Kinetic traces for the MQ^•+^ formation (at 550 nm) recorded in the presence of the lowest MQ^**+**^ concentration of 1 mM in water/acetonitrile solution buffered at pH = 6.7 (50 mM phosphate buffer), at 25 °C, and a pressure range from 5 to 60 MPa. The fit of the kinetic data to a single exponential function (y = A*exp{-(x-x0)/t}+y0) results in the values of k_obs-ET_ at each pressure. Source data

Source Data Extended Data Fig. 3 Datasets for kinetic traces (Abs versus time) at pressures of 5, 30 and 60 MPa.

**Extended Data Fig. 4:**
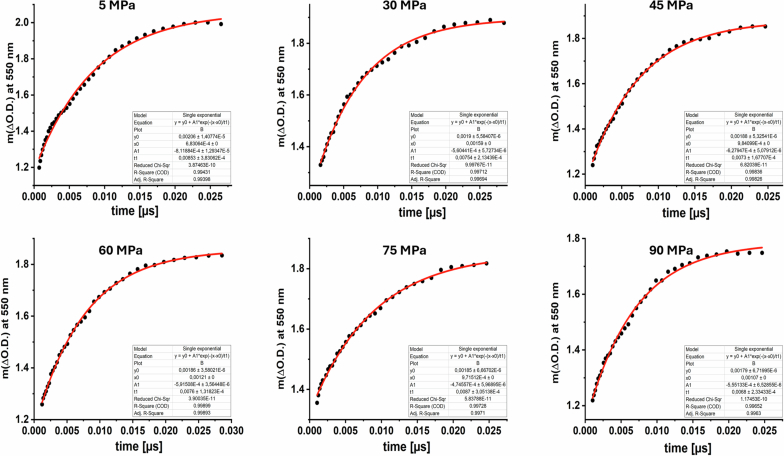
Determination of pseudo-first order rate constants (_kobs-CPET_) for the *CPET reaction between *[Ru LH]^2+^ and MQ^+^. Kinetic traces for the MQ^•+^ formation (at 550 nm) recorded in the presence of the highest MQ^+^ concentration of 100 mM in water/acetonitrile solution buffered at pH = 6.7 (50 mM phosphate buffer), at 25 °C, and a pressure range from 5 to 90 MPa. The fit of the kinetic data to a single exponential function (y = A*exp{-(x-x0)/t}+y0) results in the values of k_obs-CPET_ at each pressure. Source data

**Extended Data Fig. 6:**
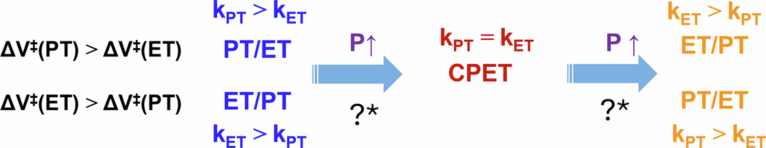
Modulations of PCET mechanisms by pressure. *Further investigation of stepwise and concerted PCET processes at elevated pressures, supported by theoretical studies, is necessary to identify potential mechanistic changes induced by pressure.

Source Data Extended Data Fig. 4 Datasets for kinetic traces (Abs versus time) in the pressure range of 5–90 MPa.

## Figures and Tables

**Extended Data Fig. 5:**
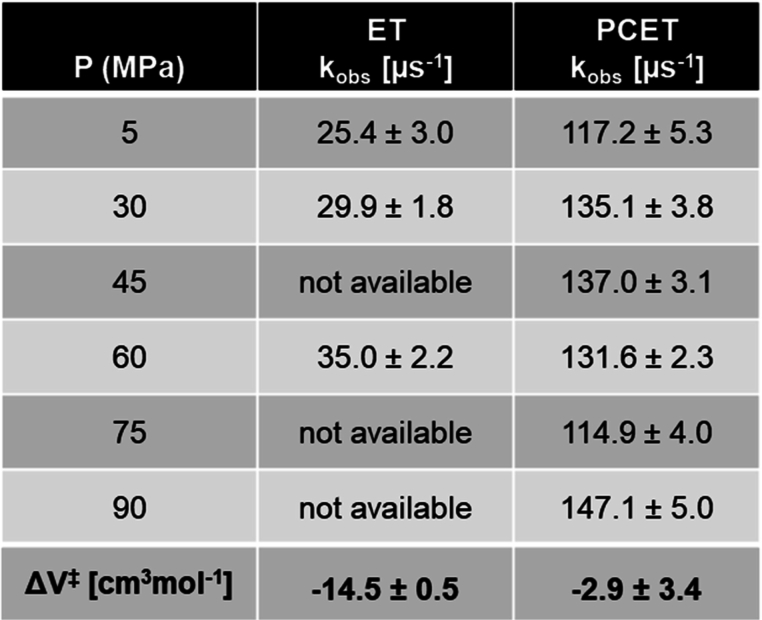
Pressure dependence of k_obs_ and the values of activation volumes obtained for the *ET and *CPET reactions. The values of k_obs_ at each pressure were obtained as described in Extended Data Figs. 3 and 4. The activation volumes for *ET and *CPET reactions were determined based on the pressure dependence of ln(k_obs-ET_) and k_obs-CPET_) as shown in Fig. 6b and d.

## Data Availability

The authors declare that the data supporting the findings of this study are available within the paper and its Supplementary Information. All raw data are available via figshare at 10.6084/m9.figshare.27063286 (ref. ^48^). Source data are provided with this paper.
