# Publisher Correction: High-pressure pump–probe experiments reveal the mechanism of excited-state proton-coupled electron transfer and a shift from stepwise to concerted pathways

**DOI:** 10.1038/s41557-025-01812-0

**Published:** 2025-03-28

**Authors:** Daniel Langford, Robin Rohr, Stefan Bauroth, Achim Zahl, Alicja Franke, Ivana Ivanović-Burmazović, Dirk M. Guldi

**Affiliations:** 1 FAU Profile Center Solar, Department of Chemistry and Pharmacy and Interdisciplinary Center for Molecular Materials (ICMM), Friedrich-Alexander-Universität Erlangen-Nürnberg, Erlangen, Germany; 2 Department of Chemistry and Pharmacy, Friedrich-Alexander-Universität Erlangen-Nürnberg, Erlangen, Germany; 3 Department of Chemistry, Ludwig-Maximilian-Universität München, Munich, Germany

**Keywords:** Electron transfer, Inorganic chemistry, Reaction kinetics and dynamics, Excited states

Correction to: *Nature Chemistry* 10.1038/s41557-025-01772-5, published online 20 March 2025.

In the version of the article initially published, the wrong Supplementary information was included. This has now been replaced, and the correct Supplementary information can be found online.

## Supplementary information

Supplementary Information

