# Supplementary material for: High-pressure pump–probe experiments reveal the mechanism of excited-state proton-coupled electron transfer and a shift from stepwise to concerted pathways
Source: Nat Chem. 2025 Mar 20;17(6):847–55. doi: 10.1038/s41557-025-01772-5 (PMC12141043; doi:10.1038/s41557-025-01772-5)
Supplement: Supplementary file 1 — Supplementary Figs. 1.1, 2.1-2.2, 3.1-3.13 and 4.1-4.14, Tables 3.1 and 3.2, Discussion and Kinetic analysis. [file 41557_2025_1772_MOESM1_ESM.pdf]

nature chemistry

Supplementary information https://doi.org/10.1038/s41557-025-01772-5

High-pressure pump–probe experiments reveal the mechanism of excited-state proton-coupled electron transfer and a shift from stepwise to concerted pathways

In the format provided by the authors and unedited

Table of Content

1 Pressure Apparatus 3

2 Steady-State Emission and Absorption Experiments 4

3 Transient Absorption Spectroscopy and kinetic studies in the absence of MQ^+ 6

3.1 Phosphate Buffered System at pH 6.7 in the absence of MQ^+ 6

3.2 Britton-Robinson Buffered System at Various pHs in the absence of MQ^+ 7

3.3 Kinetic analysis of k_{obs1}, k_{obs2} and k_{obs3} as a function of buffer concentration and pressure without MQ^+ 14

3.4 Target Analysis without MQ^+ at various Phosphate Buffer Concentrations 20

3.4.1 [Ru] (0.2 mM), phosphate (5 mM) 20

3.4.2 [Ru] (0.2 mM), phosphate (0.5 mM) 21

3.5 Target Analysis without MQ^+ at various PIPES Buffer Concentrations 21

3.5.1 [Ru] (0.2 mM), PIPES (0.5 mM) 21

3.5.2 [Ru] (0.2 mM), PIPES (5 mM) 22

3.5.3 [Ru] (0.2 mM), PIPES (50 mM) 23

4 TAS and kinetic analysis of the date collected in the presence of MQ^+ 24

4.1 Phosphate Buffered System 24

4.1.1 [Ru] (0.2 mM), MQ^+ (1 mM), phosphate (50 mM) 24

4.1.2 [Ru] (0.2 mM), MQ^+ (100 mM), phosphate (50 mM) 25

4.1.3 [Ru] (0.2 mM), MQ^+ (100 mM), phosphate (0.5 mM) 28

4.1.4 [Ru] (0.2 mM), MQ^+ (100 mM), phosphate (5 mM) 29

4.1.5 Protonation of $MQ^{\bullet+}$ and Thermal Reverse PCET 30

4.2 PIPES Buffered System 33

4.2.1 [Ru] (0.2 mM), MQ^+ (100 mM), PIPES (50 mM) 34

4.2.2 [Ru] (0.2 mM), MQ^+ (100 mM), PIPES (5 mM) 35

4.2.3 [Ru] (0.2 mM), MQ^+ (100 mM), PIPES (0.5 mM) 35

4.3 Target Analysis in the presence of various MQ^+ concentrations at 50 mM Pipes Buffer ____ 36

4.3.1 [Ru] (0.2 mM), MQ^+ (10 mM), PIPES (50 mM) ____ 36

4.3.2 [Ru] (0.2 mM), MQ^+ (1 mM), PIPES (50 mM) ____ 36

5 References ____ 37

1 Pressure Apparatus

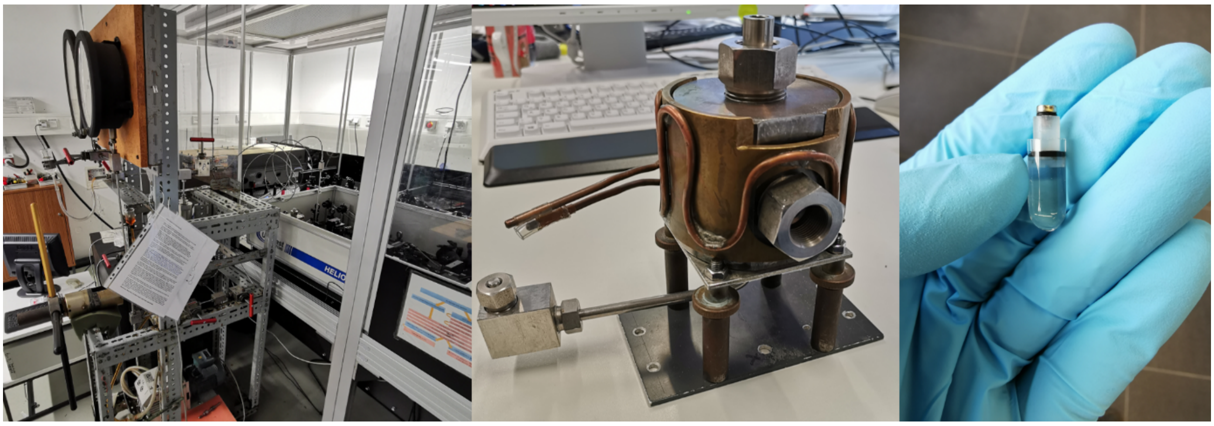

Supplementary Fig. 1.1. Experimental setup for pressure dependent TA experiments. Left: Pressure pump in front of the HELIOS spectrometer. The pressure built up by turning the wheel is transferred through the coil to the pressure cell with water as the pressure medium. Center: Pressure cell. The coil coming from the pump is connected at the left. The pump/probe pulses pass the cell through the optical windows at the front and backside. The top screw gives access to the sample chamber. Right: Cuvette used for pressure dependent TA experiments.

2 Steady-State Emission and Absorption Experiments

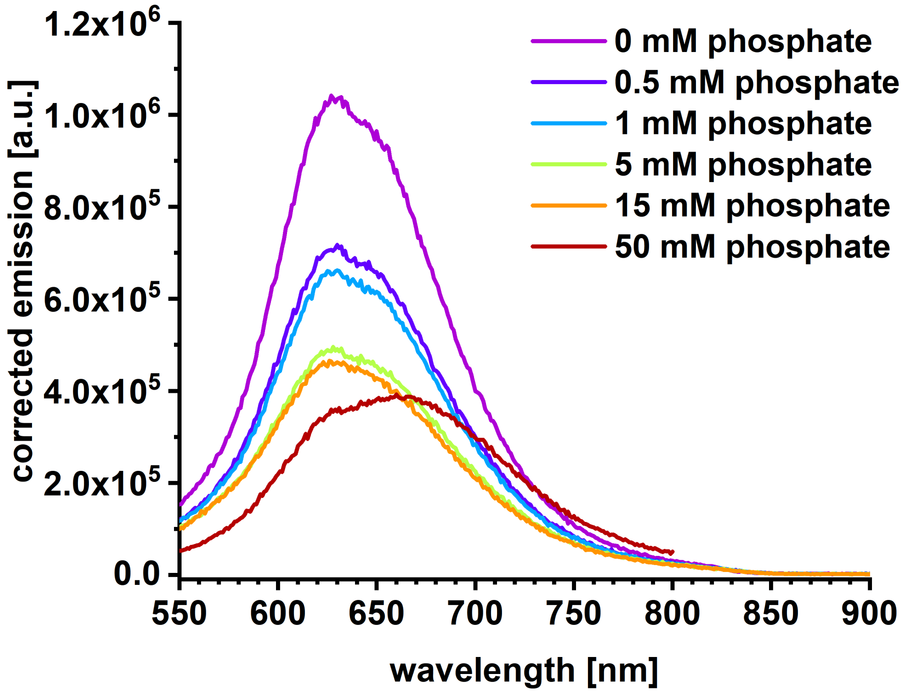

Supplementary Fig. 2.1. Emission spectra were recorded at room temperature in acetonitrile/water 1:1 (v/v) with the following composition: c($[Ru(bpy)_2pyimH]^{2+}$) = 0.2 mM, c(MQ^+) = 100 mM and various phosphate concentrations at a pH of 6.7. Photoexcitation at 473 nm slitwidth of 5 nm for excitation and emission, and 0.2 s integration time.

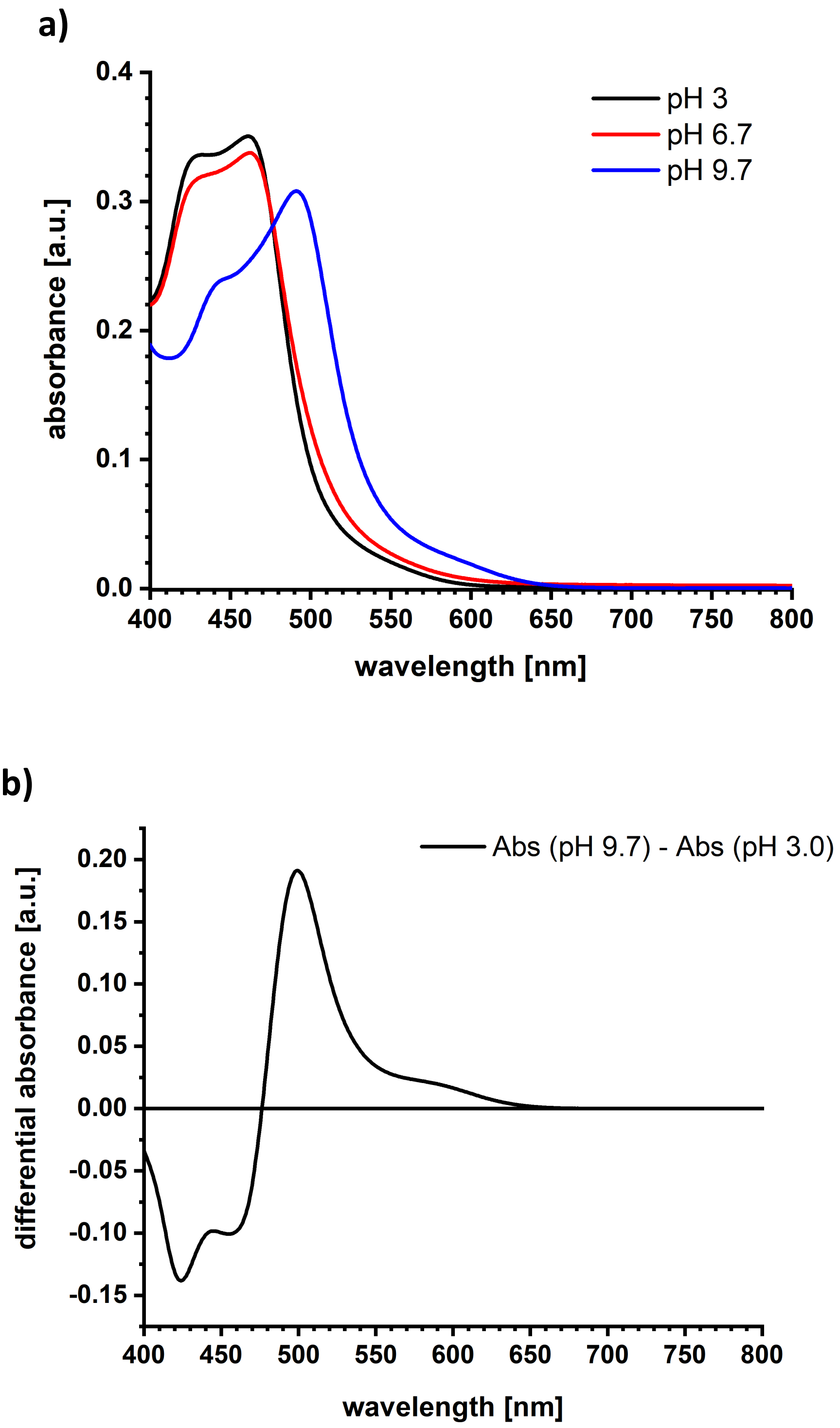

Supplementary Fig. 2.2. a, Absorption spectra of $[Ru(bpy)_2pyimH]^{2+}$ (0.2 mM) in acetonitrile/water 1:1 (v/v) with 50 mM Britton-Robinson buffer at various pHs. The spectra were recorded in a cuvette with optical pathlength of 4 mm. **b**, Difference of ground state UV-vis spectra of deprotonated and protonated complex, i.e., Abs(**$[Ru\text{-}L]^{+}$**) – Abs(**$[Ru\text{-}LH]^{2+}$**).

3 Transient Absorption Spectroscopy and kinetic studies in the absence of MQ^+

3.1 Phosphate Buffered System at pH 6.7 in the absence of MQ^+

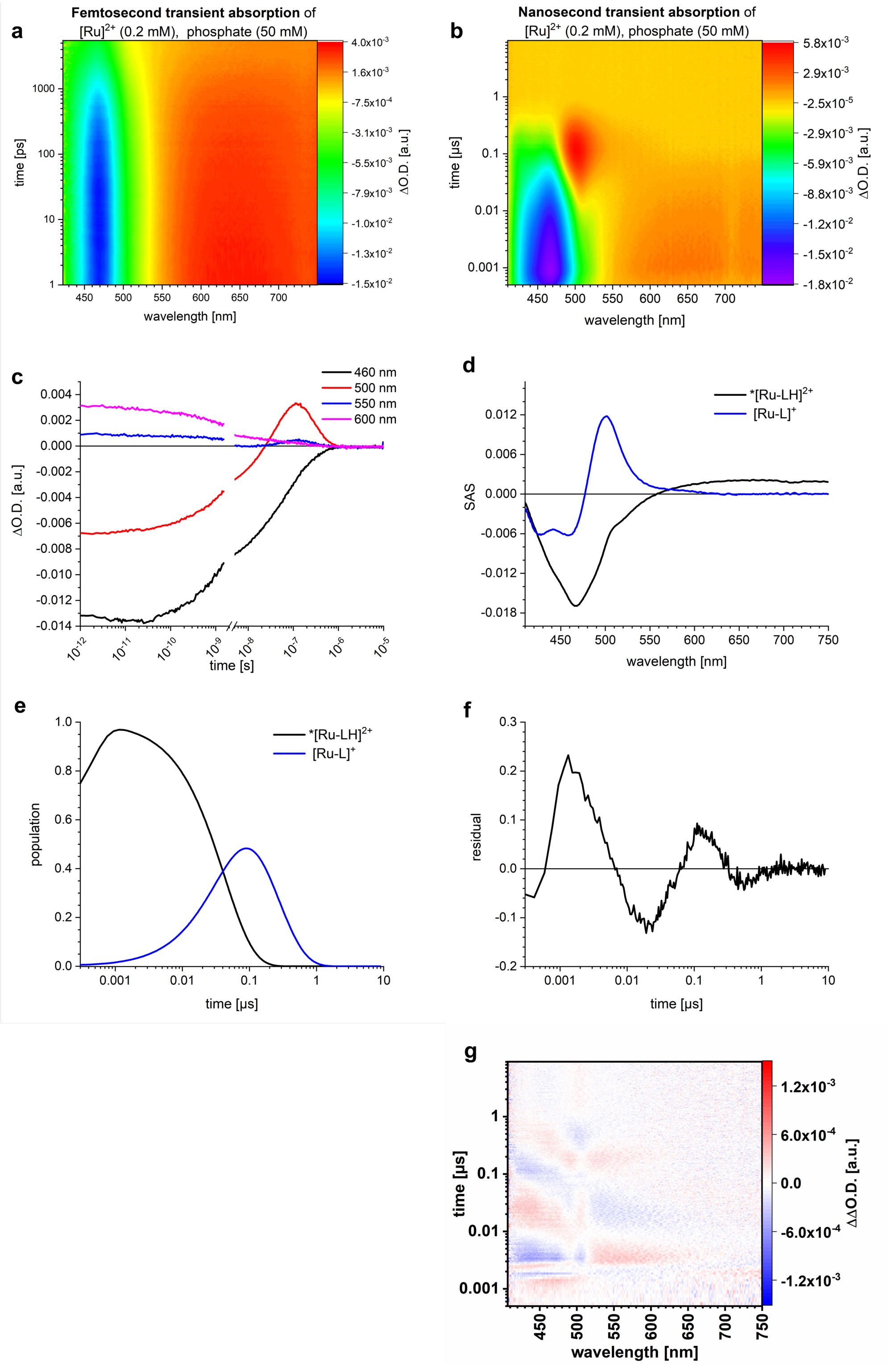

Supplementary Fig. 3.1. a, Zero point and chirp corrected fs-TAS spectra. **b,** Zero point and chirp corrected ns-TAS spectra. **c,** Time traces from fs- and ns-TAS spectra at 460 (black), 500 (red), 550 (blue) and 600 nm (purple). **d,** Species associated

spectra (SAS) from biexponential target analysis of the ns-TAS spectra, recorded for $[Ru(bpy)_2pyimH]^{2+}$ in phosphate buffer at pH of 6.7. **e,** Time evolution of the species associated spectra (SAS) from target analysis of the ns-TAS spectra. **f,** Residual trace and **g**, 2D differential residual map (below) of the ns-TAS spectra shown in **a** after biexponential fitting with target analysis, demonstrating low quality of the fit. All spectra were recorded at room temperature in acetonitrile/water 1:1 (v/v) with the following composition: c($[Ru(bpy)_2pyimH]^{2+}$) = 0.2 mM, c(phosphate) = 50 mM. The time traces on the early (1 ps-1 ns) and late time scale (1 ns-350 μs) were measured with different spectrometers, hence the discrepancy of the ΔO.D. values.

3.2 Britton-Robinson Buffered System at Various pHs in the absence of MQ^+

To confirm the occurrence of the intermediate step involving the deprotonation in the excited state, i.e., the conversion of **$*[Ru\text{-}LH]^{2+}$** to **$*[Ru\text{-}L]^{+}$** under mid-range pH, we conducted additional experiments to characterize the spectral properties in both its ground and excited states at three different pHs, that is, 3.0, 6.7, and 9.7, using a universal Britton-Robinson buffer to avoid changes in the chemical identity of the buffer.

As illustrated in Supplementary Fig. 2.2a, steady-state absorption spectra reveal distinct spectral features at the selected pHs. At an acidic pH of 3.0, the ground-state exhibits two absorption peaks at 430 and 460 nm, corresponding to the protonated **$[Ru\text{-}LH]^{2+}$** form. In contrast, at a basic pH of 9.7, the absorptions at 445 and 490 nm are attributed to the deprotonated **$[Ru\text{-}L]^{+}$** form. At a neutral pH of 6.7, absorptions at 430 and 460 nm indicate the presence of the protonated **$[Ru\text{-}LH]^{2+}$** form, while a weak shoulder between 475 and 630 nm suggests a minor presence of the deprotonated **$[Ru\text{-}L]^{+}$** species. These observations are consistent with the previously reported pK_a^0 of 8.1 ± 0.1 and the known ground-state characteristics.[1]

The fs- and ns-TAS experiments provided insights into the spectral characteristics of the excited state under conditions of the selected pHs. At a pH of 3.0, 387 nm photoexcitation of **$[Ru\text{-}LH]^{2+}$** resulted in a broad excited-state absorption (ESA) between 508 and 750 nm and ground-state bleaching (GSB) at 428 and 461 nm (Supplementary Fig. 3.2). The protonated excited species **$*[Ru\text{-}LH]^{2+}$** decays monoexponentially to the ground-state **$[Ru\text{-}LH]^{2+}$** within 127 ns (Supplementary Fig. 3.2f). The EAS spectrum obtained from the global analysis of the ns-TAS spectrum shown in Supplementary Fig. 3.2d corresponds to the differential absorption spectrum of the protonated excited state species, **$*[Ru\text{-}LH]^{2+}$**, and the protonated ground state species, **$[Ru\text{-}LH]^{2+}$**. At a pH of 9.7, the 387 nm photoexcitation of **$[Ru\text{-}L]^{+}$** generated ESA in the range from 578 to 750 nm along with GSB at 445 and 490 nm (Supplementary Fig. 3.3). The deprotonated excited state **$*[Ru\text{-}L]^{+}$** decays monoexponentially with a lifetime of 41.7 to the deprotonated ground state **$[Ru\text{-}L]^{+}$** (Supplementary Fig. 3.3f**)**. Accordingly, global analysis of the TAS spectra resulted in the EAS spectrum shown in Supplementary Fig. 3.3d which can be

attributed to the differential absorption spectrum of the deprotonated excited species, ***[Ru-L]$^+$,** and the deprotonated ground state species, **[Ru-L]**$^+$.

Thus, under conditions of a sufficiently low pH (i.e., pH < pK_a* < pK_a^0), where deprotonation of the excited state does not occur, or a sufficiently high pH (i.e., pK_a* < pK_a^0 < pH), where both ground and excited state species are deprotonated, only a single-step transition between two species, i.e. monoexponential decay from ***[Ru-LH]$^{2+}$** to **[Ru-LH]$^{2+}$** and monoexponential decay from ***[Ru-L]**$^+$ to **[Ru-L]**$^+$, respectively, are observed.

Quite a different scenario is observed upon photoexcitation at a pH of around 6.7, where the starting ground state of the complex is in the protonated form, whereas the excited state is expected to undergo deprotonation (pK_a* < pH < pK_a^0). In this case, 387 nm photoexcitation of **[Ru-LH]$^{2+}$** led to a broad ESA in the range from 545 to 750 nm and GSB at 422 and 466 nm (***[Ru-LH]$^{2+}$**), followed by the decay of the ESA above 545 nm with the concomitant formation of a prominent 500 nm ESA (Supplementary Fig. 3.4). Formation of the latter points to the transient accumulation of the deprotonated form **[Ru-L]**$^+$ based on the similarity of this band to that seen for independently prepared difference spectrum of the deprotonated **[Ru-L]**$^+$ and protonated **[Ru-LH]$^{2+}$** ground states (Supplementary Fig. 2.2b). A two-exponential fit describing a two-step process, that is, a conversion from the protonated excited species ***[Ru-LH]$^{2+}$** to the deprotonated electronic ground state **[Ru-L]**$^+$, prior to reprotonation to recover the starting condition **[Ru-LH]$^{2+}$**, resulted in a poor fit with significant residual traces (Supplementary Fig. 3.5). As shown in Supplementary Fig. 3.4, only the use of a three-exponential fit resulted in good quality of the fit. Photoexcitation gave rise to the protonated excited form ***[Ru-LH]$^{2+}$**. The ^{3}MLCT state was then found to undergo deprotonation to result in ***[Ru-L]**$^+$, as a superposition of the ^{3}MLCT ESA from ***[Ru-LH]$^{2+}$** together with the 507 nm ESA from the deprotonated **[Ru-L]**$^+$. Subsequently, the deprotonated electronic ground state is **[Ru-L]**$^+$, before reprotonation to recover the starting form **[Ru-LH]$^{2+}$** occurs. As such, three SAS (Supplementary Fig. 3.4d**)** from which the first one (black line) is attributed to the the protonated excited state and the protonated ground state, i.e. ***[Ru-LH]$^{2+}$** and **[Ru-LH]$^{2+}$** (note the similarity of this differential spectrum to that observed under the conditions of pH 3), the second one (red line) is ascribed to ***[Ru-L]**$^+$, whereas third SAS (blue line) represents the deprotonated **[Ru-L]**$^+$ and protonated **[Ru-LH]$^{2+}$** ground states as described above.

At pH 6.7, the formation of the deprotonated excited species ***[Ru-L]**$^+$ is based on the fact that photoexcitation of its protonated ground state at this pH (pK_a^0 = 8.1) will induces deprotonation of the initial excited state based on a much lower value of pK_a* equal to 5.3. According to three-

exponential analysis the deprotonated **$*[Ru\text{-}L]^+$** species is formed within 25.7 ns to from **$*[Ru\text{-}LH]^{2+}$** and is characterized by the additional ESA at 507 nm. In the next reaction step, **$*[Ru\text{-}L]^+$** decays within 45.6 ns to the deprotonated, ground state **$[Ru\text{-}L]^+$** reflected in the formation of prominent 500 nm feature. The following protonation of the ground state **$[Ru\text{-}L]^+$** to recover **$[Ru\text{-}LH]^{2+}$** takes place within 293.7 ns and is spectroscopically identified by the simultaneous decay of the 500 nm ESA and with the repopulation of the 422 and 460 nm GSBs.

In summary, the additional fs- and ns-TAS experiments performed in Britton-Robinson buffer at three different pHs clearly point out that in the middle pH range, the deprotonation event in the excited state, i.e. the transition from **$*[Ru\text{-}LH]^{2+}$** to **$*[Ru\text{-}L]^+$** is observed and is followed kinetically using the method described in this study. Not only do the consistent spectral features related to **$*[Ru\text{-}L]^+$** support its nature as a deprotonated excited state, but this is also corroborated by the similar decay lifetimes determined in four independent experiments and atributed to the **$*[Ru\ L]^+$** $\rightarrow$ **$[Ru\ L]^+$** transformation. As stated above, experiments were conducted at a pH of either 9.7 or 6.7 in Britton-Robinson buffer, yielding lifetimes of 41.7 and 45.6 ns, respectively. The remaining two experiments were performed at a pH of 6.7 in phosphate buffer, resulting in lifetimes of 47 ± 5 ns in the absence of **MQ^+** (based on k_2 = 21 ± 2 μs^{-1}, see Extended Figure 1) and 35 ± 2 ns in the presence of **MQ^+** (based on the intercept $k_{4\text{-}p}$ = 28.7 ± 1.8 μs^{-1}, which is related to the parallel intramolecular quenching **$*[Ru\ L]^+$** $\rightarrow$ **$[Ru\ L]^+$**; see Fig. 5h in the main text).

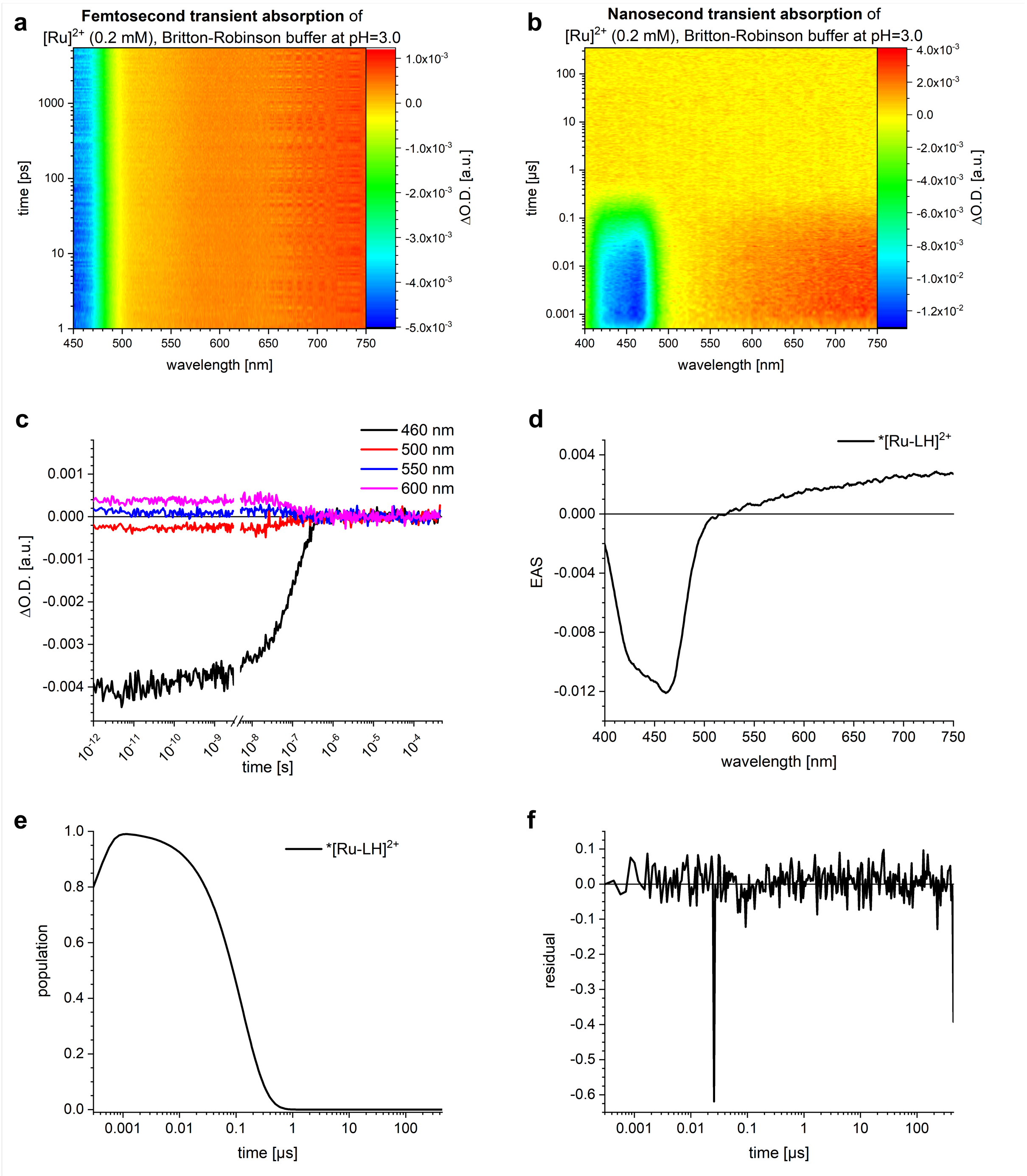

Supplementary Fig. 3.2. a, Zero point and chirp corrected fs-TAS spectra. **b,** Zero point and chirp corrected ns-TAS spectra. **c,** Time traces from fs- and ns-TAS spectra at 460 (black), 500 (red), 550 (blue), and 600 nm (purple). **d,** Evolution associated spectra (ESA) from monoexponential global analysis of the ns-TAS spectra, the black line represents the differential spectrum of ***[Ru-LH]**$^{2+}$ formed after the excitation and the starting **[Ru-LH]**$^{2+}$complex. **e,** Time evolution of the evolution associated spectra (ESA) from monoexponential global analysis of the ns-TAS spectra. **f,** Residual of the ns-TAS spectra after monoexponential fitting with global analysis, demonstrating the good quality of the fit. All spectra were recorded at room temperature in acetonitrile/water 1:1 (v/v) with the following composition: c([Ru(bpy)$_2$pyimH]$^{2+}$) = 0.2 mM and c(buffer) = 50 mM at pH = 3.0. The time traces on the (1 ps-1 ns) time scale and on the (1 ns-350 μs) time scale were measured with different spectrometers, hence the discrepancy of the ΔO.D. values.

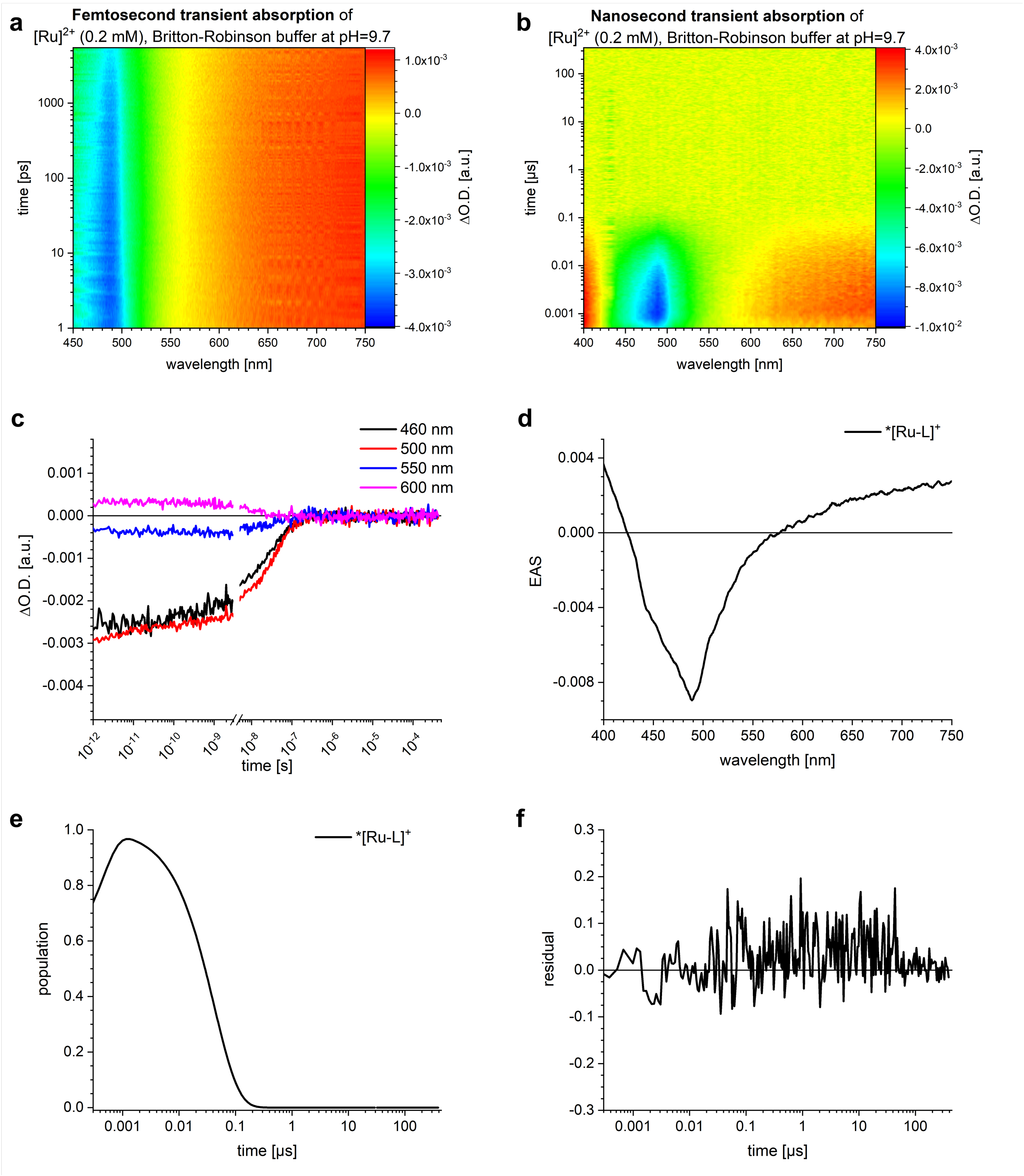

Supplementary Fig. 3.3. a, Zero point and chirp corrected fs-TAS spectra. **b,** Zero point and chirp corrected ns-TA spectra. **c,** Time traces from fs- and ns-TAS spectra at 460 (black), 500 (red), 550 (blue), and 600 nm (purple). **d,** Evolution associated spectra (EAS) from monoexponential global analysis of the ns-TAS spectra, the black line represents the differential spectrum of ***[Ru-L]**$^+$formed after the excitation and the starting **[Ru-L]**$^+$ complex. **e,** Time evolution of the evolution associated spectra (EAS) from monoexponential global analysis of the ns-TAS spectra. **f,** Residual of the ns-TAS spectra after monoexponential fitting with global analysis, demonstrating the good quality of the fit. All spectra were recorded at room temperature in acetonitrile/water 1:1 (v/v) with the following composition: c($[Ru(bpy)_2pyimH]^{2+}$) = 0.2 mM and c(buffer) = 50 mM at pH = 9.7. The time traces on the (1 ps-1 ns) time scale and on the (1 ns-350 µs) time scale were measured with different spectrometers, hence the discrepancy of the ΔO.D. values.

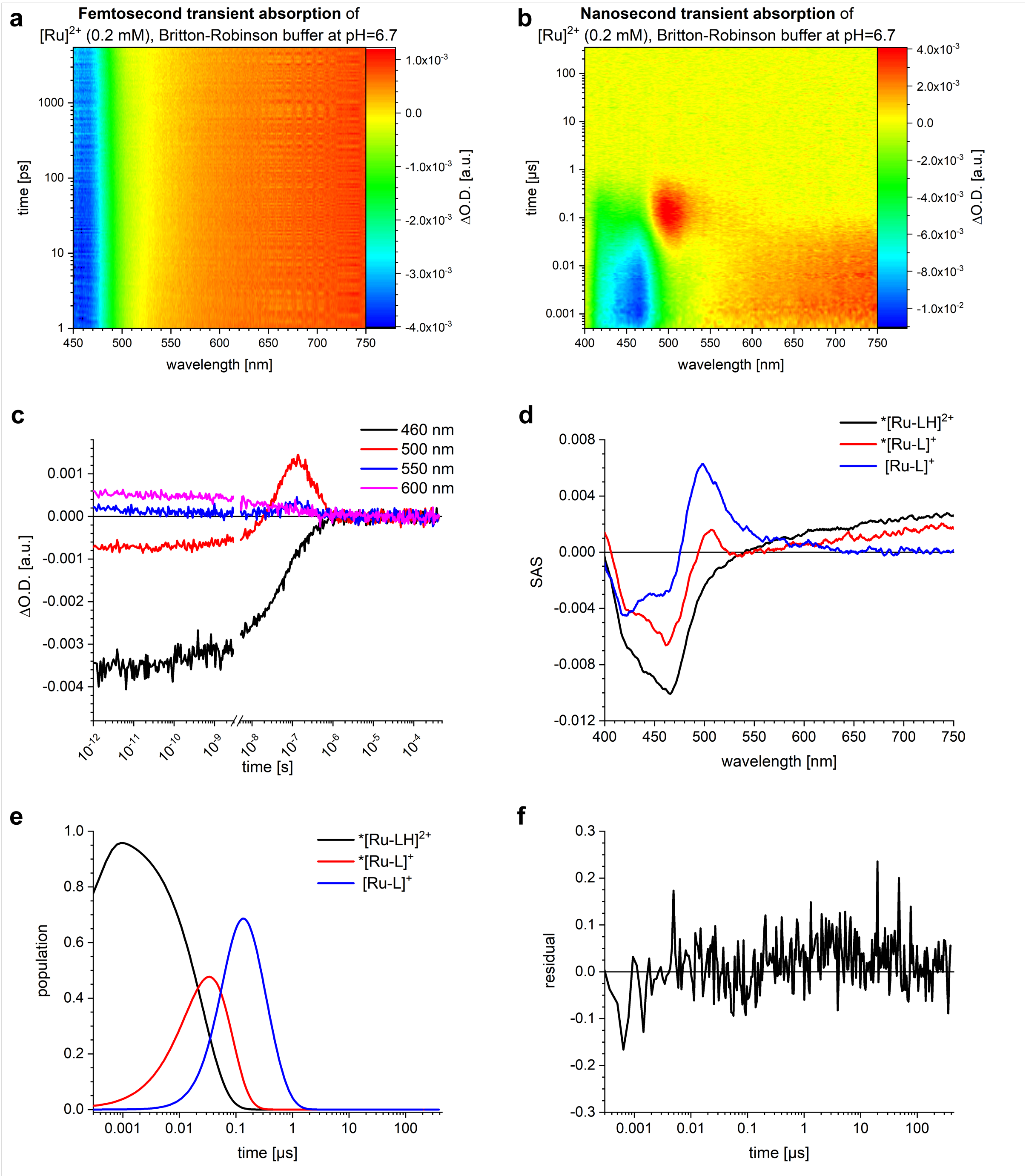

Supplementary Fig. 3.4. a, Zero point and chirp corrected fs-TAS spectra. **b,** Zero point and chirp corrected ns-TAS spectra. **c,** Time traces from fs- and ns-TAS spectra at 460 (black), 500 (red), 550 (blue), and 600 nm (purple). **d,** Species associated spectra (SAS) from three-exponential target analysis of the ns-TAS spectra, the black line represents the differential spectrum of **$*[Ru\text{-}LH]^{2+}$** and the starting **$[Ru\text{-}LH]^{2+}$**, the red line describes the differential spectrum of **$*[Ru\text{-}L]^{+}$** and the starting **$[Ru\text{-}LH]^{2+}$** and blue line depicts the differential spectrum of **$[Ru\text{-}L]^{+}$** and the starting **$[Ru\text{-}LH]^{2+}$**. **e,** Time evolution of the species associated spectra (SAS) from target analysis of the ns-TAS spectra. **f,** Residual of the ns-TAS spectra after three-exponential fitting with target analysis, demonstrating the good quality of the fit. All spectra were recorded at room temperature in acetonitrile/water 1:1 (v/v) with the following composition: c($[Ru(bpy)_2pyimH]^{2+}$) = 0.2 mM and c(buffer) = 50 mM at pH = 6.7. The time traces on the (1 ps-1 ns) time scale and on the (1 ns-350 µs) time scale were measured with different spectrometers, hence the discrepancy of the ΔO.D. values.

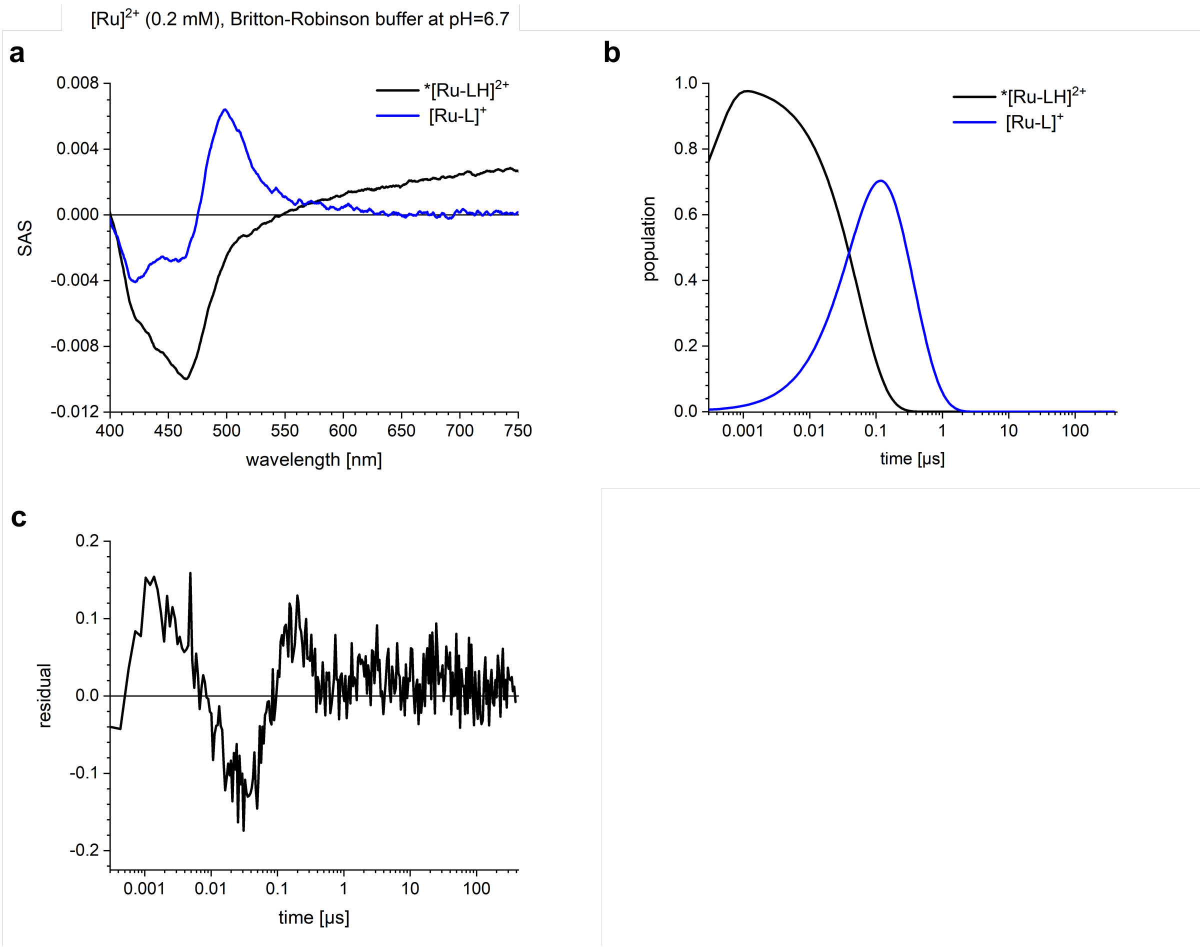

Supplementary Fig. 3.5. a, Species associated spectra from biexponential target analysis of the ns-TAS spectra, recorded from $[Ru(bpy)_2pyimH]^{2+}$ in Britton-Robinson buffer at a pH of 6.7. **b,** Time evolution of the species associated spectra (SAS) from target analysis of the ns-TAS spectra. **c,** Residual of the ns-TAS spectra after biexponential fitting with target analysis, demonstrating insufficient quality of the fit. All spectra were recorded at room temperature in acetonitrile/water 1:1 (v/v) with the following composition: c($[Ru(bpy)_2pyimH]^{2+}$) = 0.2 mM and c(buffer) = 50 mM. The time traces on the (1 ps-1 ns) time scale and on the (1 ns-350 µs) time scale were measured with different spectrometers, hence the discrepancy of the ΔO.D. values.

3.3 Kinetic analysis of k_{obs1}, k_{obs2} and k_{obs3} as a function of buffer concentration and pressure without MQ^+

The existence of the pre-equilibrium involving first complex formation with the buffer components is common in the case of buffer catalysis and for **Eq. 1** and **3** including HPO_4^{2-} or $H_2PO_4^-$ as reaction partners, (Fig. 4a in the main text and Supplementary Fig. 3.8a), the deprotonation/protonation reaction steps can be expressed as demonstrated in Eq.4 and Eq.5, respectively.

$$\text{*}\mathbf{Ru^{2+}\text{-}LH} + HPO_4^{2-} \rightleftarrows \text{*}\mathbf{Ru^{2+}\text{-}L^-} + H_2PO_4^- \qquad \text{(Eq. 1)}$$

$$\text{*}\mathbf{Ru^{2+}\text{-}L^-} \longrightarrow \mathbf{Ru^{2+}\text{-}L^-} \qquad \text{(Eq. 2)}$$

$$\mathbf{Ru^{2+}\text{-}L^-} + H_2PO_4^- \rightleftarrows \mathbf{Ru^{2+}\text{-}LH} + HPO_4^{2-} \qquad \text{(Eq. 3)}$$

$$\text{*}\mathbf{Ru^{2+}\text{-}LH} + HPO_4^{2-} \overset{K_1}{\rightleftarrows} \{\text{*}\mathbf{Ru^{2+}\text{-}LH}\text{–}HPO_4^{2-}\}$$

$$\text{*}\mathbf{Ru^{2+}\text{-}LH} \xrightarrow[+\,H_2O]{k_{1\text{-}p}} \text{*}\mathbf{Ru^{2+}\text{-}L^-} + H_3O^+ \qquad \{\text{*}\mathbf{Ru^{2+}\text{-}LH}\text{–}HPO_4^{2-}\} \xrightarrow{k_1} \text{*}\mathbf{Ru^{2+}\text{-}L^-} + H_2PO_4^-$$

$$k_{obs1} = (k_{1\text{-}p} + k_1K_1[HPO_4^{2-}])/(1 + K_1[HPO_4^{2-}]) \qquad \text{(Eq. 4)}$$

$$\mathbf{Ru^{2+}\text{-}L^-} + H_2PO_4^- \overset{K_3}{\rightleftarrows} \{\mathbf{Ru^{2+}\text{-}L^-}\text{–}H_2PO_4^-\}$$

$$\mathbf{Ru^{2+}\text{-}L^-} \xrightarrow[+\,H_2O]{k_{3\text{-}p}} \mathbf{Ru^{2+}\text{-}LH} + OH^- \qquad \{\mathbf{Ru^{2+}\text{-}L^-}\text{–}H_2PO_4^-\} \xrightarrow{k_3} \mathbf{Ru^{2+}\text{-}LH} + HPO_4^{2-}$$

$$k_{obs3} = (k_{3\text{-}p} + k_3K_3[H_2PO_4^-])/(1 + K_3[H_2PO_4^-]) \qquad \text{(Eq. 5)}$$

The occurrence of significant intercept in the buffer concentration dependence of k_{obs1} and k_{obs3} suggests the involvement of the parallel reaction ($k_{1\text{-}p}$ or $k_{3\text{-}p}$) in the decay kinetics leading to the same deprotonation/protonation product. Since it is known that decay of the excited state of [**Ru^{2+}-LH**] also proceeds in aqueous solution in the absence of any buffer it is supposed that parallel reaction seen in the present study can be ascribed to the reaction with water molecules as potential proton acceptor/donor as shown in Eqs. 4 and 5. If so, the observed rate constants

measured for the deprotonation and protonation reactions, k_{obs1} and k_{obs3} can be expressed as in **Eq. 4** and **Eq. 5**, respectively. The values of appropriate rate/equilibrium constants obtained from the fit of experimental data to **Eq. 4** and **5** for the first and third reaction as well as the values of activation/reaction volumes for the respective steps of the deprotonation/protonation reactions are reported in the Extended Data Figure 2.

The buffer concentration dependence and saturation kinetics observed for the second reaction (**Eq. 2**, Supplementary Fig. 3.7a) can be explained in term of the fact that ***Ru**$^{2+}$**-L**$^{-}$ being the only reactant in **Eq. 2** is generated in the phosphate-involving equilibrium **Eq. 1**, thus, the observed rate constants (k_{obs2}) will increase with increasing [HPO_4^{2-}] until the saturation concentration of ***Ru**$^{2+}$**-L**$^{-}$ in **Eq. 1** is reached (k_{obs2} = k_2[***Ru**$^{2+}$**-L**$^{-}$]). In this context, for this reaction step only pressure dependence of k_{obs2} at the highest buffer concentration as well as the parallel reaction (decay of excited state in the absence of buffer, $k_{2\text{-}p}$) was analyzed as shown in Supplementary Fig. 3.7b,c.. The appropriate values of activation volume determined for this reaction are summarized in the Extended Figure 1. .

For the bimolecular reaction with the reactants **A** and **B** and product **AB**, the conceivable mechanism is formulated according to the transition state theory as shown in Eq. 6.

$$A + B \rightleftarrows (A{-}{-}{-}B)^{\ddagger} \longrightarrow AB \qquad \text{Eq. 6}$$

The overall partial molar volume change, $\Delta V°$, and the volume of activation, $\Delta V^{\ddagger}$, for this reaction is expressed as follows (Eq. 7 and Eq. 8, respectively):

$$\Delta V° = V_{AB} - V_A - V_B \qquad \text{Eq. 7}$$

$$\Delta V^{\ddagger} = V^{\ddagger}_{(A{-}{-}{-}B)} - V_A - V_B \qquad \text{Eq. 8}$$

where V_A, V_B, V_{AB} and $V^{\ddagger}_{(A{-}{-}{-}B)}$ correspond to the partial molar volumes of the reactants, the product, and the transition state, respectively. The magnitude and sign of the overall partial molar volume change, $\Delta V°$, and of the volume of activation, $\Delta V^{\ddagger}$, depend on the nature of the chemical species involved and their evironmental stabilization.

The value of $\Delta V°$ is determined directly by dilatometry or estimated from a combination of the partial molar volumes of the reactants and product according to the Eq. 6. In the thermodynamic/kinetic investigations, the reaction volume is often derived from the pressure dependence of the equilibrium constant (K) measured at constant temperature (T) according to

Eq. 9, whereas the volume of activation is only determined from the effect of pressure on the rate constant (k) of the reaction at a constant temperature (T) (Eq. 10).

$$[\delta \ln(K)/\delta P)]_T = - \Delta V°/RT \qquad \text{Eq. 9}$$

$$[\delta \ln(k)/\delta P]_T = - \Delta V^{‡}/RT \qquad \text{Eq. 10}$$

As described above, kinetic analysis of k_{obs1}, k_{obs2} and k_{obs3} as a function of buffer concentration and pressure without MQ^+ revealed saturation behaviour indicating the occurrence of the pre-equilibrium with parallel reactions, that is, k_{obs1} and k_{obs3} as shown in Eqs. 4 and 5. In such case, the effect of pressure on the equilibrium constants, K_1 and K_3, the first-order rate constants, k_1 and k_3, as well as the first-order rate constants for the parallel reactions, $k_{1\text{-}p}$ and $k_{3\text{-}p}$ are resolved by investigating the buffer concentration dependence of k_{obs1} and k_{obs3} at various pressure and considering the conditions of limited range of buffer concentration (a – c) as described below and shown in Supplementary Fig. 3.6 on an example of the first reaction step (k_{obs1}).

a) Under conditions of very low buffer concentration ([buffer]~ 0 M), the expressions for k_{obs1} or k_{obs3} (Eqs. 4 and 5, respectively) are simplified to $k_{obs1} = k_{1\text{-}p}$ or $k_{obs3} = k_{3\text{-}p}$ and the respective values can be determined from the intercept of the buffer concentration dependence at every individual pressure. With the values of $k_{1\text{-}p}$ or $k_{3\text{-}p}$ at each pressure at hand, the volume of activation for the parallel reactions, i.e. $\Delta V^{‡}(k_{1\text{-}p})$ or $\Delta V^{‡}(k_{3\text{-}p})$, are calculated from the slope of the pressure dependence of $\ln(k_{1\text{-}p})$ or $\ln(k_{3\text{-}p})$ (Fig. 4d in the main text and Supplementary Fig. 3.8d) according to Eq. 10.

b) Under conditions of low buffer concentration, i.e. in the concentration range, where the plateau has not yet been reached, the expressions for k_{obs1} or k_{obs3} is simplified to $k_{obs1} = k_{1\text{-}p} + k_1K_1[\text{buffer}]$ and $k_{obs3} = k_{3\text{-}p} + k_3K_3[\text{buffer}]$. In other words, the slope of the linear fit of k_{obs1} or k_{obs3} on buffer concentration results in values of k_1K_1 and k_3K_3 at each measured pressure. From the slope of the linear dependence of $\ln(k_1K_1)$ or $\ln(k_3K_3)$ on pressure (Fig.4c in the main text and Supplementary Fig. 3.8c) the values of $\Delta V^{‡}(k_1K_1)$ or $\Delta V^{‡}(k_3K_3)$ are determined according to Eq. 10. Note that $\Delta V^{‡}(k_1K_1)$ or $\Delta V^{‡}(k_3K_3)$ is expressed as $\Delta V^{‡}(k_1K_1) = \Delta V°(K_1) + \Delta V^{‡}(k_1)$ or $\Delta V^{‡}(k_3K_3) = \Delta V°(K_3) + \Delta V^{‡}(k_3)$.

c) Under conditions of high buffer concentration, i.e. in the region of saturation, the expressions for k_{obs1} and k_{obs3} are simplified to $k_{obs1} = k_1$ or $k_{obs3} = k_3$. Measuring the values of k_1 or k_3 under such conditions at each pressure enables to determining $\Delta V^{‡}(k_1)$ or $\Delta V^{‡}(k_3)$ from the slope of the linear fits of $\ln(k_1)$ or $\ln(k_3)$ on pressure (Eq. 10, Fig. 4b in the main text and Supplementary Fig. 3.8b). Having both $\Delta V^{‡}(k_1K_1)$ and $\Delta V^{‡}(k_1)$ or $\Delta V^{‡}(k_3K_3)$ and $\Delta V^{‡}(k_3)$, the values of

$\Delta V°(K_1)$ or $\Delta V°(K_3)$ are calculated via $\Delta V°(K_1) = \Delta V^‡(k_1K_1) - \Delta V^‡(k_1)$ or $\Delta V°(K_3) = \Delta V^‡(k_3K_3) - \Delta V^‡(k_3)$.

For the reason described above, in the case of the second reaction step (Eq. 2) only pressure dependence of k_{obs2} at the highest buffer concentration (i.e. under the conditions described in c)) as well as the pressure dependence of the parallel reaction, k_{2-p} (i.e. under the conditions described in a)) were considered to determine $\Delta V^‡(k_2)$ and $\Delta V^‡(k_{2-p})$, respectively (Supplementary Fig. 3.7b,c).

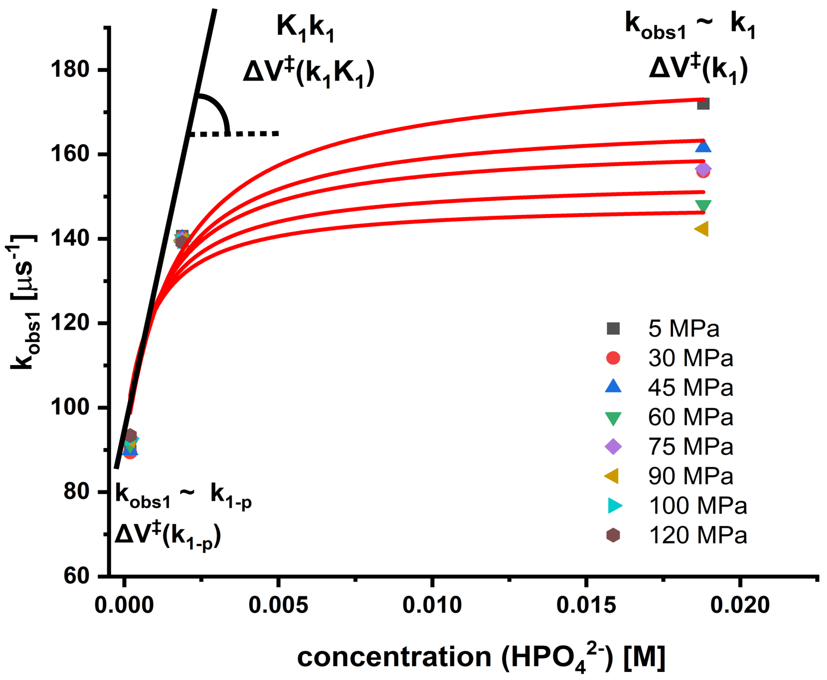

Supplementary Fig. 3.6. Treatment of the pressure- and buffer concentration dependent data to resolve reaction and activation volume for the appropriate equilibria/reactions as described in the subsections **a-c** using the example of the first reaction (k_{obs1}).

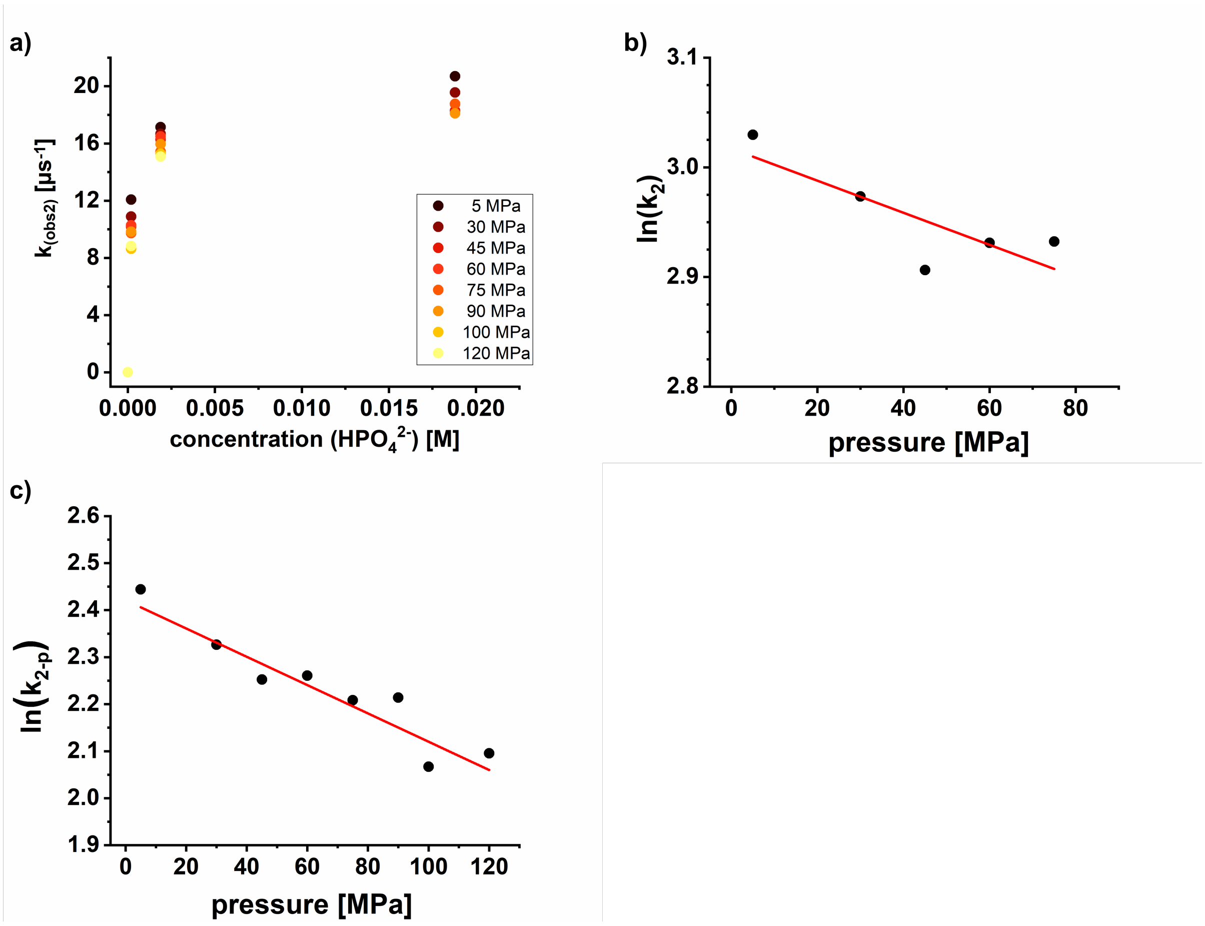

Supplementary Fig. 3.7. a, Dependence of k_{obs2} on buffer concentration ([HPO_4^{2-}]) and pressure (5 – 120 MPa) measured for the second reaction step in a phosphate buffer at a pH of 6.7 and T = 298 K. **b** and **c**, Pressure dependences of ln(k_2) and ln(k_{2-p}), respectively, to determine the values of activation volume for the respective reactions. The parameters for the linear fits depicted in **b** and **c** are summarized in Supplementary Table 3.1.

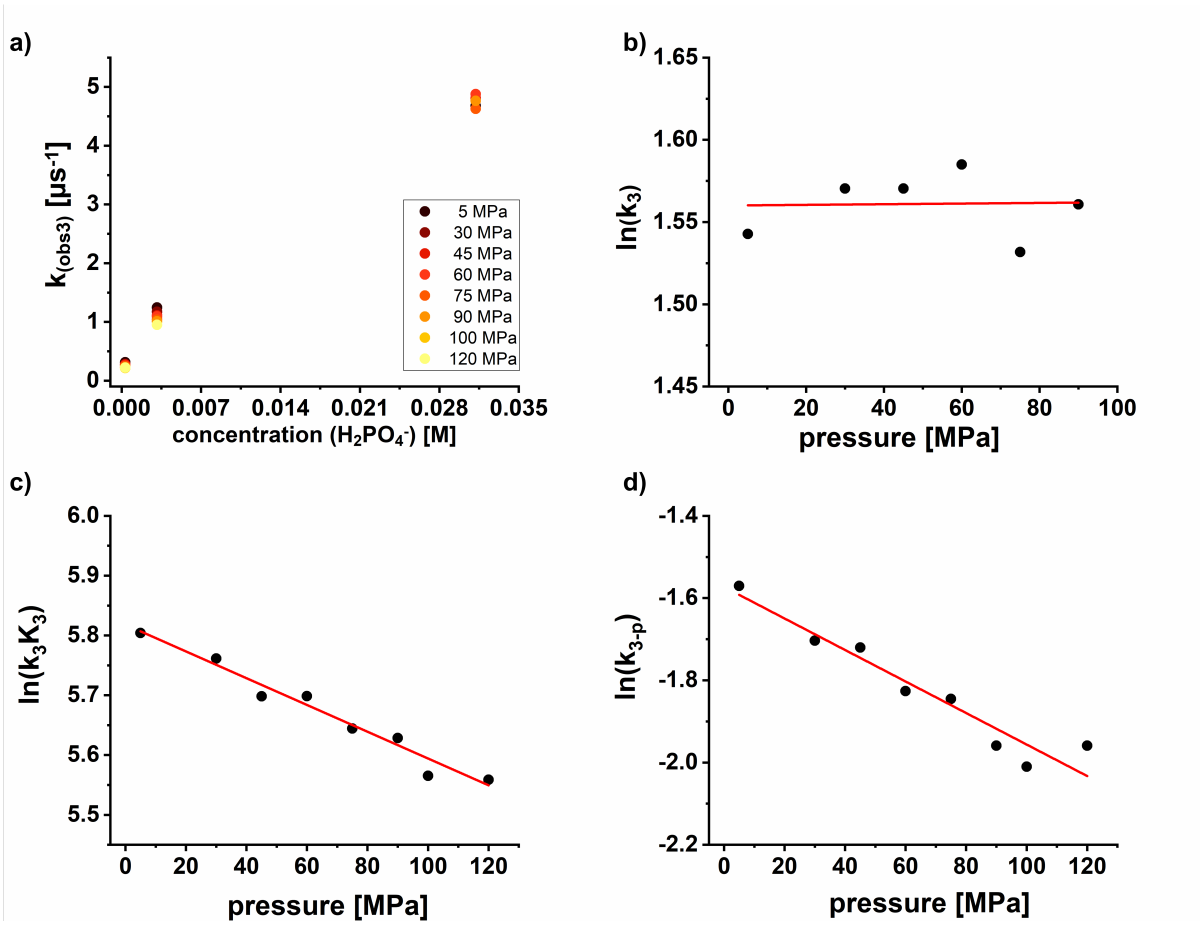

Supplementary Fig. 3.8. a, Dependence of k_{obs3} on buffer concentration ($[H_2PO_4^-]$) and pressure (5 – 120 MPa) measured for the third reaction step in a phosphate buffer at a pH of 6.7 and T = 298 K. **b** - **d** Pressure dependences of $\ln(k_3)$, $\ln(k_3K_3)$ and $\ln(k_{3\text{-}p})$ to determine the values of activation volume for the respective reactions. The parameters for the linear fits depicted in **b** - **d** are summarized in Supplementary Table 3.1.

Supplementary Table 3.1 Parameters of the linear fits to experimental data shown in Figures 4b-d, 5h, 6b, 6d in the main text and Supplementary Figures 3.7b-c, 3.8b-d and 4.9.

	Output parameters for linear fit $y = a + bx$					
	a	b	R^2	Adj. R^2	RSS	Pearson's r
Figure 4b	$(-15 \pm 4.1) \times 10^{-4}$	5.12 ± 0.02	0.6925	0.6412	11.05	-0.8321
Figure 4c	$(-3.8 \pm 6.3) \times 10^{-4}$	10.3 ± 0.03	0.0667	-0.1199	5.642	-0.2583
Figure 4d	$(11.6 \pm 3.2) \times 10^{-4}$	4.40 ± 0.02	0.6874	0.6353	6.680	0.8291
Supp. Figure 3.7b	$(-14.6 \pm 5.9) \times 10^{-4}$	3.02 ± 0.02	0.6751	0.5667	9.835	-0.8216
Supp. Figure 3.7c	$(-30.1 \pm 3.9) \times 10^{-4}$	2.42 ± 0.02	0.9094	0.8942	10.38	-0.9536
Supp. Figure 3.8b	$(0.2 \pm 3.3) \times 10^{-4}$	1.56 ± 0.01	8.7×10^{-4}	-0.2489	26.21	0.0294
Supp. Figure 3.8c	$(-22.4 \pm 1.4) \times 10^{-4}$	5.82 ± 0.01	0.9770	0.9731	1.669	-0.9884
Supp. Figure 3.8d	$(-38.3 \pm 4.6) \times 10^{-4}$	-1.57 ± 0.03	0.9220	0.9090	2.368	-0.9602
Figure 5h	965.5 ±52.6	29.04 ± 1.82	0.9970	0.9941	0.286	0.9985
Figure 6b	$(57.8 \pm 1.8) \times 10^{-4}$	3.20 ± 0.01	0.9991	0.9981	0.007	0.9995
Figure 6d	$(11.2 \pm 14.2) \times 10^{-4}$	4.81 ± 0.09	0.1335	-0.0831	4.788	0.3654
Supp. Figure 4.9	232.5 ± 11.9	2.11 ± 0.14	0.9974	0.9948	0.248	0.9987

3.4 Target Analysis without MQ^+ at various Phosphate Buffer Concentrations

3.4.1 [Ru] (0.2 mM), phosphate (5 mM)

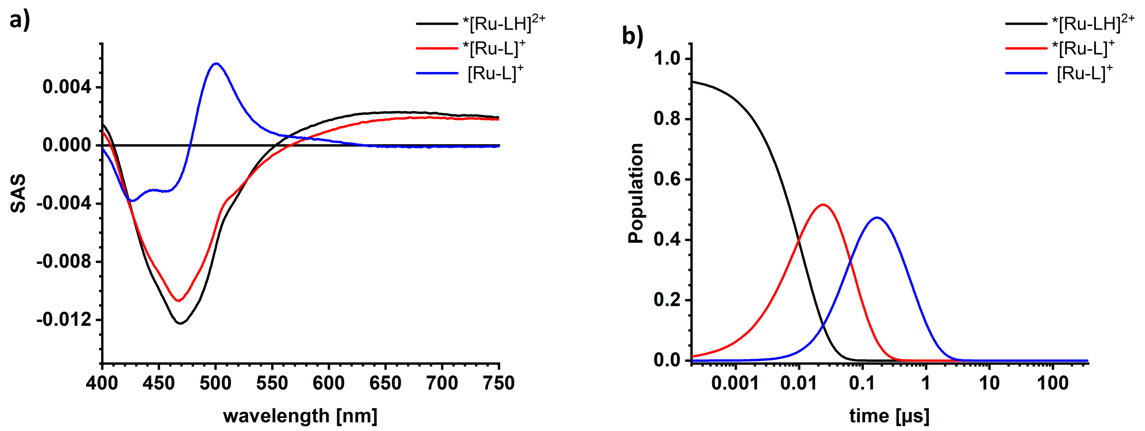

Supplementary Fig. 3.9. a, Species associated spectra (SAS) from target analysis of c(Ru) = 0.2 mM and c(phosphate) = 5 mM in acetonitrile/water 1:1 (v/v). **b,** Corresponding time-population profiles of the different SAS.

3.4.2 [Ru] (0.2 mM), phosphate (0.5 mM)

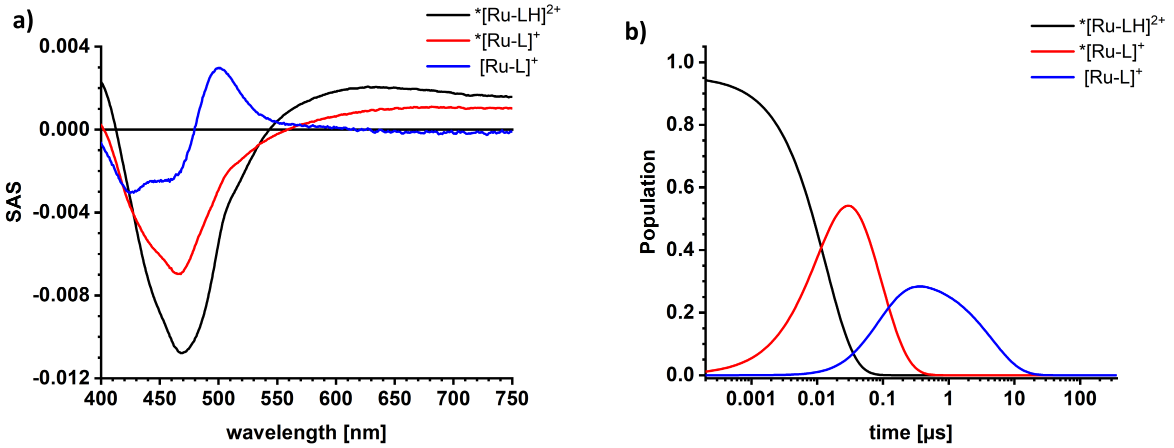

Supplementary Fig. 3.10. a, Species associated spectra (SAS) from target analysis of c(Ru) = 0.2 mM and c(phosphate) = 0.5 mM in acetonitrile/water 1:1 (v/v). **b**, Corresponding time-population profiles of the different SAS (Fig.2b in the main text).

3.5 Target Analysis without MQ^+ at various PIPES Buffer Concentrations

3.5.1 [Ru] (0.2 mM), PIPES (0.5 mM)

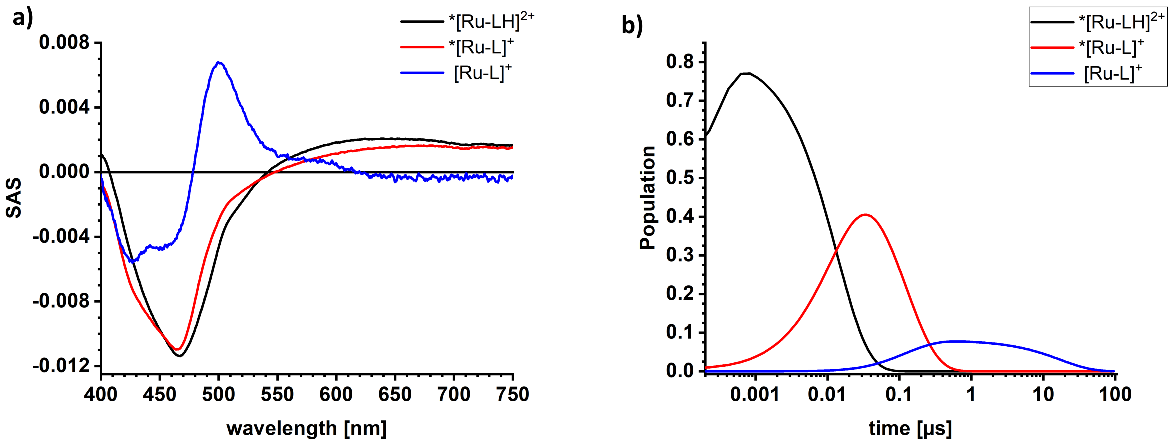

Supplementary Fig. 3.11. a, Species associated spectra (SAS) from target analysis of c(Ru) = 0.2 mM and c(PIPES) = 0.5 mM in acetonitrile/water 1:1 (v/v). **b**, Corresponding time-population profiles of the different SAS.

3.5.2 [Ru] (0.2 mM), PIPES (5 mM)

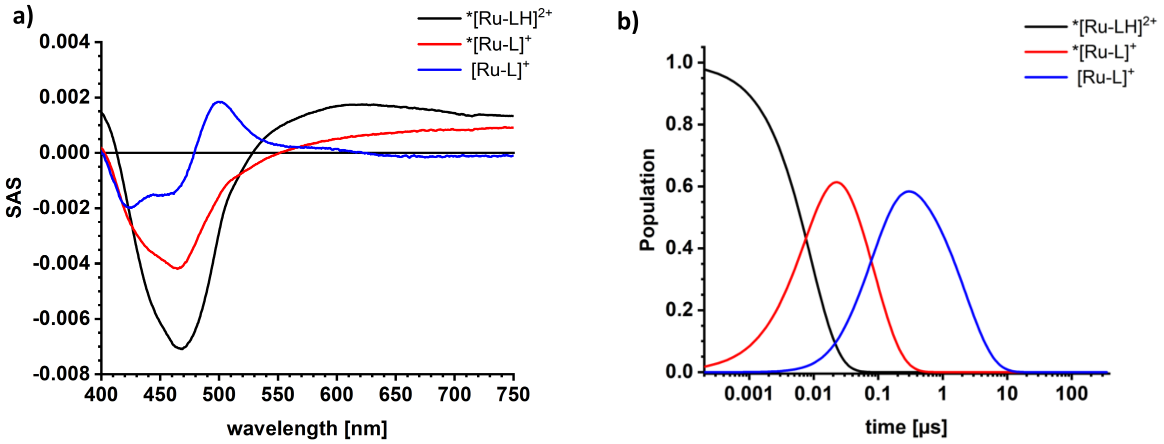

Supplementary Fig. 3.12. a, Species associated spectra (SAS) from target analysis of c(Ru) = 0.2 mM and c(PIPES) = 5 mM in acetonitrile/water 1:1 (v/v). **b**, Corresponding time-population profiles of the different SAS.

3.5.3 [Ru] (0.2 mM), PIPES (50 mM)

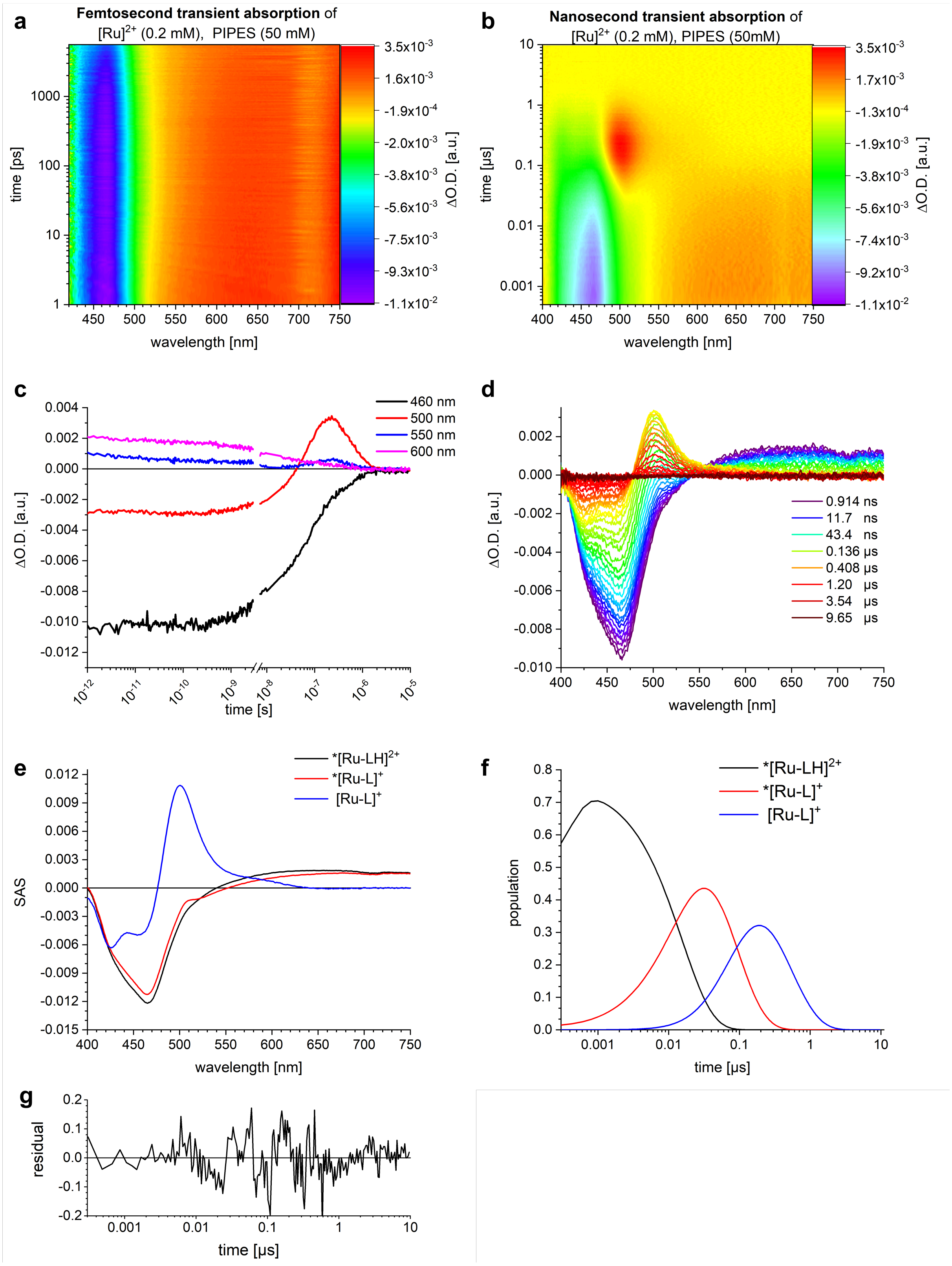

Supplementary Fig. 3.13. **a**, Zero point and chirp corrected fs-TAS spectra. **b**, Zero point and chirp corrected ns-TA spectra. **c**, Time traces from fs- and ns-TAS spectra at 460 (black), 500 (red), 550 (blue), and 600 nm (purple). **d**, Spectral slices from the ns-TAS spectra. **e**, Species associated spectra (SAS) from target analysis of the ns-TA spectra. **f**, Time evolution of the species associated spectra (SAS) from target analysis of the ns-TAS spectra. **g**, Residual of the ns-TAS spectra after fitting with

target analysis. All spectra were recorded at room temperature in acetonitrile/water 1:1 (v/v) with the following composition: c([Ru(bpy)$_2$pyimH]$^{2+}$) = 0.2 mM and c(PIPES) = 50 mM. The time traces on the (1 ps-1 ns) time scale and on the (1 ns-350 μs) time scale were measured with different spectrometers, hence the discrepancy of the ΔO.D. values.

Supplementary Table 3.2 Summary of the rate constants, equilibrium constants with PIPES buffer components and corresponding activation and reaction volumes, $\Delta V^{\ddagger}$ and $\Delta V°$, respectively, for the decay reaction steps of photoexcited *Ru^{2+}-LH state in the absence of electron accepting MQ^+.[a]

*[Ru-LH]$^{2+}$ ⟶ *[Ru-L]$^{+}$		*[Ru-L]$^{+}$ ⟶ [Ru-L]$^{+}$		[Ru-L]$^{+}$ ⟶ [Ru-LH]$^{2+}$		Unit
k_1	70.1 ± 36	k_2	12.4 ± 5	k_3	2.1 ±1.1	μs^{-1}
k_1K_1	12 ± 3	k_2K_2	-	k_3K_3	0.2 ± 0.05	nM^{-1}s^{-1}
K_1	172 ± 79	K_2	-	K_3	77.1 ± 63	M^{-1}
$k_{1\text{-p}}$	75 ± 22	$k_{2\text{-p}}$	8.4 ± 3.2	$k_{3\text{-p}}$	0.03 ± 0.1	μs^{-1}
$\Delta V^{\ddagger}(k_1)$	+5.5 ± 4.6	$\Delta V^{\ddagger}(k_2)$	+1.7 ± 0.9	$\Delta V^{\ddagger}(k_3)$	+0.9 ± 0.4	cm^{3}mol^{-1}
$\Delta V^{\ddagger}(k_1K_1)$	+1.2 ± 6.3	$\Delta V^{\ddagger}(k_2K_2)$	-	$\Delta V^{\ddagger}(k_3K_3)$	+2.6 ± 0.6	cm^{3}mol^{-1}
$\Delta V°(K_1)$	-4.3 ± 10.9	$\Delta V°(K_2)$	-	$\Delta V°(K_3)$	+1.7 ± 1.0	cm^{3}mol^{-1}
$\Delta V^{\ddagger}(k_{1\text{-p}})$	-1 ± 2.9	$\Delta V^{\ddagger}(k_{2\text{-p}})$	+4.2 ± 0.5	$\Delta V^{\ddagger}(k_{3\text{-p}})$	-8.9 ± 2.2	cm^{3}mol^{-1}

[a]In general the pressure dependent data obtained for the reaction in PIPES buffer exhibit large experimental errors and therefore are not further discussed in details.

4 TAS and kinetic analysis of the date collected in the presence of MQ^+

4.1 Phosphate Buffered System

4.1.1 [Ru] (0.2 mM), MQ^+ (1 mM), phosphate (50 mM)

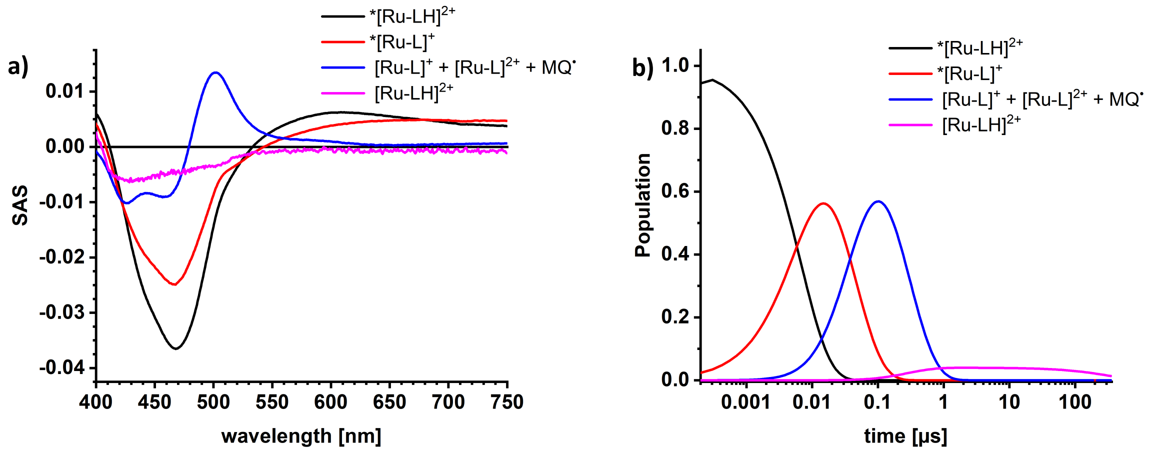

Supplementary Fig. 4.1. a, Species associated spectra (SAS) from target analysis of c(Ru) = 0.2 mM, c(MQ^+) = 1 mM and c(phosphate) = 50 mM in acetonitrile/water 1:1 (v/v) [2]. **b,** Corresponding time-population profiles of the different SAS. Due to

the low concentration of MQ^+, its protonation, i.e. generation of **$HMQ^{•+}$**, could not be resolved as a separate reaction step (for comparison see experiment with c(MQ^+) = 10 mM in Fig. 5b-c in the main text).

4.1.2 [Ru] (0.2 mM), MQ^+ (100 mM), phosphate (50 mM)

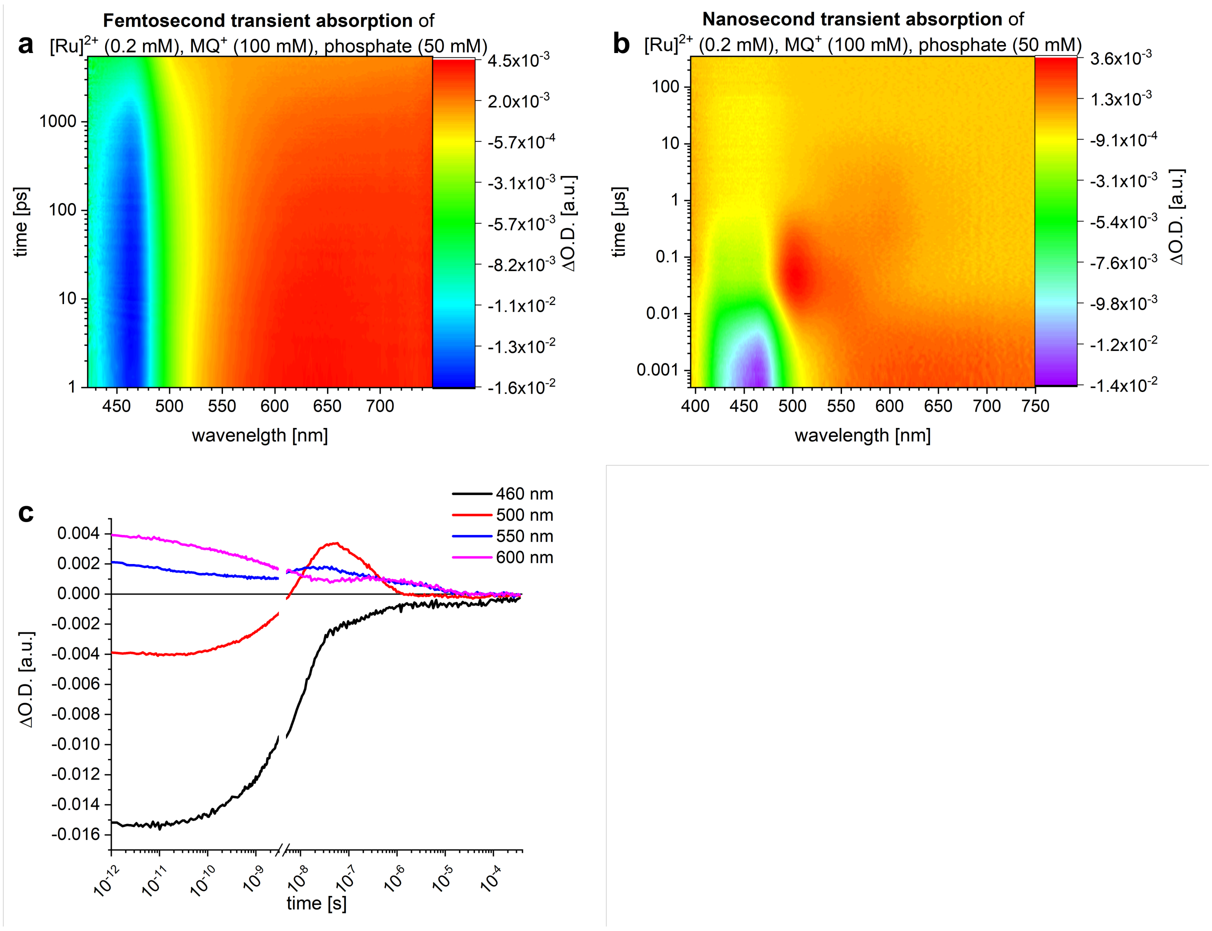

Supplementary Fig. 4.2. a, Zero point and chirp corrected fs-TAS spectra. **b**, Zero point and chirp corrected ns-TAS spectra. **c**, Time traces from fs- and ns-TAS spectra at 460 (black), 500 (red), 550 (blue), and 600 nm (purple). All spectra were recorded at room temperature in acetonitrile/water 1:1 (v/v) with the following composition: c($[Ru(bpy)_2pyimH]^{2+}$) = 0.2 mM, c(MQ^+) = 100 mM, c(phosphate) = 50 mM. The time traces on the (1 ps-1 ns) time scale and on the (1 ns-350 µs) time scale were measured with different spectrometers, hence the discrepancy of the ΔO.D. values.

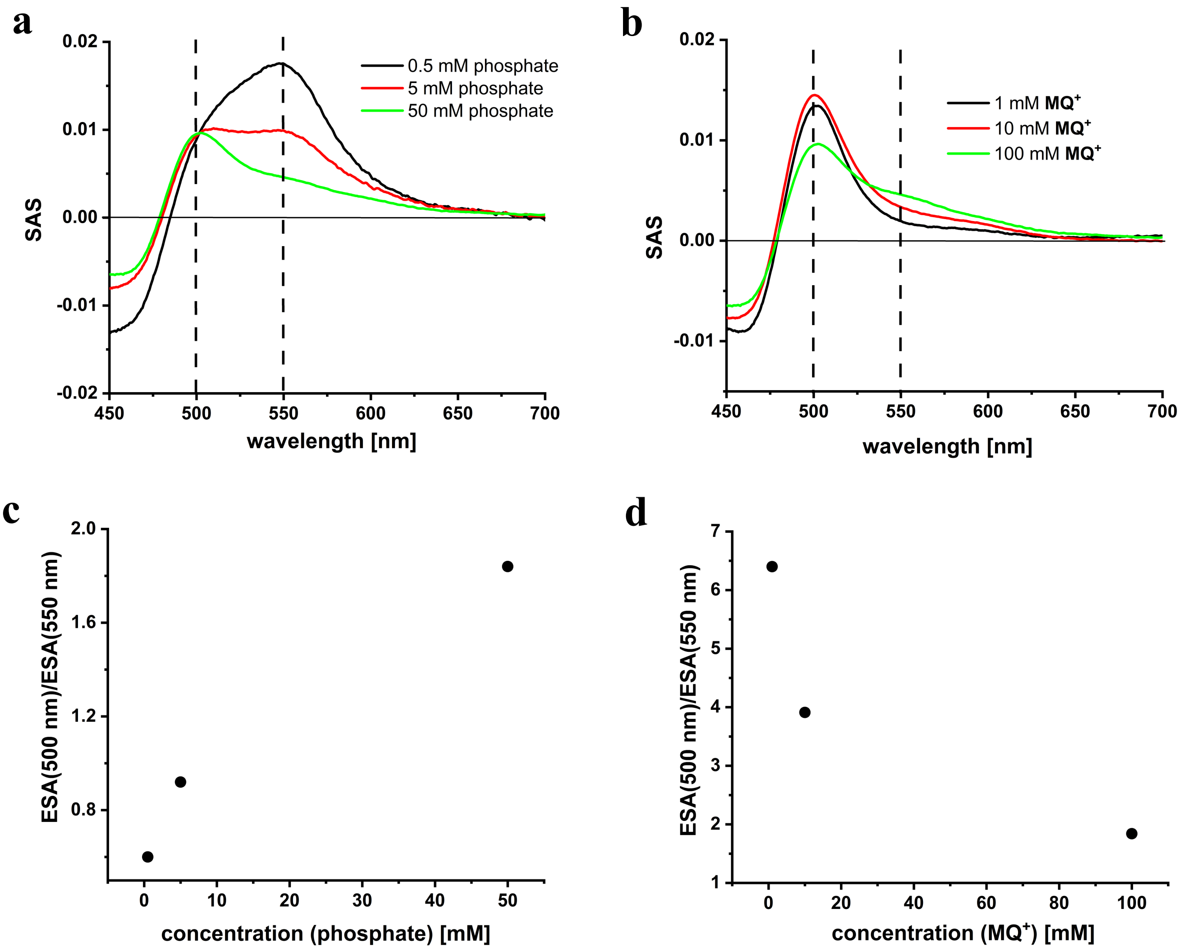

Supplementary Fig. 4.3 a, SAS after charge-seperation or intramolecular quenching, resulting in the superposition of $\mathbf{[Ru\text{-}L]^{+}}$, $\mathbf{[Ru\text{-}L]^{2+}}$, and $\mathbf{MQ^{\bullet}}$ species, obtained from target analysis of the ns-TA spectra recorded with 100 mM MQ^{+} and various phosphate buffer concentrations. **b**, SAS after charge-seperation or intramolecular quenching, resulting in the superposition of $\mathbf{[Ru\text{-}L]^{+}}$, $\mathbf{[Ru\text{-}L]^{2+}}$, and $\mathbf{MQ^{\bullet}}$ species, obtained from target analysis of the ns-TA spectra recorded in 50 mM phosphate buffer and various MQ^{+} concentratios. **c**, The ratio of ESA(500 nm)/ESA(550 nm), calculated from the data shown in **a**, based on the ESA values at 500 nm and 550 nm extracted from the SAS spectrum at the selected phosphate buffer concentration. **d**, The ratio of ESA(500 nm)/ESA(550 nm), calculated from the data shown in **b**, based on the ESA values at 500 nm and 550 nm extracted from the SAS spectrum at the selected MQ^{+} concentration.

Note: It is important to note that presented SAS represent the superposition of signals from the species $\mathbf{[Ru\text{-}L]^{+}}$, $\mathbf{[Ru\text{-}L]^{2+}}$, and $\mathbf{MQ^{\bullet}}$. This superposition arises due to the coexistence of two competing parallel processes during the corresponding reaction step:

i) Bidirectional PCET from $\mathbf{*[Ru\text{-}LH]^{2+}}$ to form $\mathbf{[Ru\text{-}L]^{2+}}$ and $\mathbf{MQ^{\bullet}}$. This process can occur either via CPET or a stepwise pathway involving $*PT_1$ followed by $*ET_1$, as shown in Fig. 1,

-Parallel intramolecular quenching pathway, where the excited state $\mathbf{*[Ru\text{-}LH]^{2+}}$ undergoes quenching to form the deprotonated ground state $\mathbf{[Ru\text{-}L]^{2+}}$ (path k_2 in Fig. 1 and Fig. 3). As explained in the manuscript (and noted in Supplementary Fig. 4.5), the absorption features

at 500 nm arise from **[Ru-L]**$^{+}$ and **[Ru-L]**$^{2+}$, with **[Ru-L]**$^{+}$ exhibiting a higher extinction coefficient than that of **[Ru-L]**$^{2+}$.[1] At 550 nm, the absorption corresponds mainly to **MQ**$^{\bullet}$. However, intensities at both wavelengths are influenced by the relative contributions of the two pathways, extinction coefficients of the observed species and the specific experimental conditions. Given these interdependencies, the ESA(500 nm)/ESA(550 nm) ratio is a more robust metric for comparing the two processes than individual intensities.

To illustrate that we can summarise the following observations in SAS intensities:

- Effect of Phosphate Buffer Concentration (Figure 4.3a): SAS intensity at 550 nm decreases with increasing phosphate concentration. Reason is that higher phosphate concentration promotes the intramolecular quenching pathway, reducing **MQ**$^{\bullet}$ formation and suppressing **[Ru-L]**$^{2+}$ generation, while enhancing **[Ru-L]**$^{+}$ formation. But no significant intensity change is observed at 500 nm because the opposing effects of reduced **[Ru-L]**$^{2+}$ and increased **[Ru-L]**$^{+}$ contributions cancel each other out. However, at high phosphate concentrations, the SAS shape resembles that of predominantly **[Ru-L]**$^{+}$.

-Effect of MQ^{+} Concentration (Figure 4.3b): SAS intensity at 550 nm increases with higher **MQ**$^{+}$ concentrations. Reason: Higher **MQ**$^{+}$ levels enhance intermolecular electron transfer resulting in more **MQ**$^{\bullet}$ and **[Ru-L]**$^{+}$. But, at 500 nm, no increase in intensity is observed because **[Ru-L]**$^{2+}$ has a lower extinction coefficient than **[Ru-L]**$^{+}$ and decreased intramolecular quenching reduces **[Ru-L]**$^{+}$ contributions (which otherwise gives more significant rise in 500 nm feature). A noticeable drop in 500 nm intensity is observed only at very high **MQ**$^{+}$ concentrations (e.g., 100 mM), where the bidirectional PCET dominates (77% contribution). For comparison at 1 mM and 10 mM **MQ**$^{+}$ the bidirectional PCET contributes only 3.2% and 25%, respectively. These contributions are based on comparison of $k_{obs(MQ+)}$ and k_2 we obtained for the bidirectional PCET and paraller intramolecular quenching, respectively.

Thus, the ESA(500 nm)/ESA(550 nm) ratio more directly, reflects the competition between the two pathways, then individual SAS intensities, as illustrated in Supplementary Figure 4.3c and 4.3d, where: Higher ratios signify a dominance of the intramolecular quenching pathway and lower ratios indicate increased contributions from bidirectional PCET, respectively.

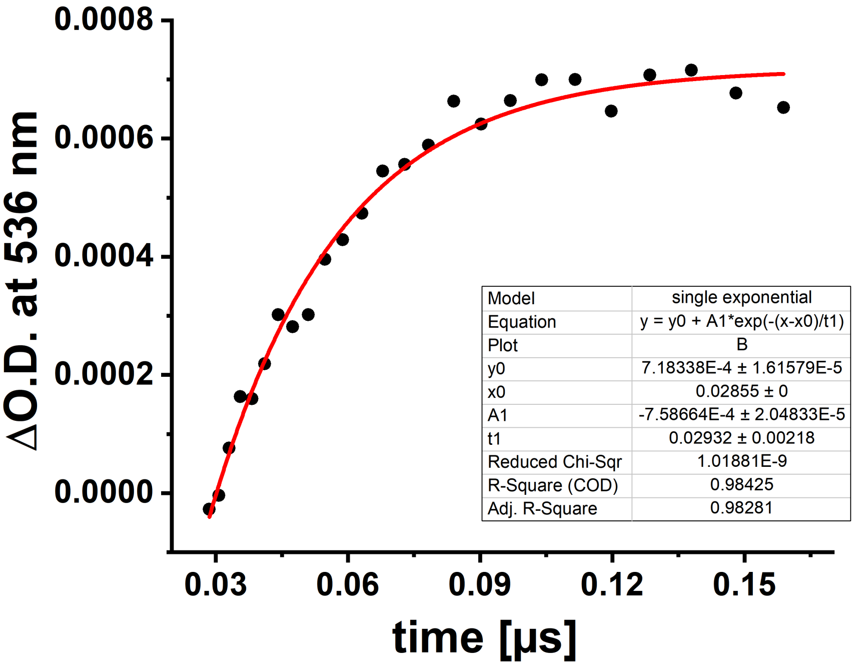

Supplementary Fig. 4.4. Kinetic trace recorded at 536 nm for the $MQ^{\bullet}$ formation under conditions of c(Ru) = 0.2 mM, c(MQ^+) = 1 mM, and c(phosphate) = 50 mM in acetonitrile/water 1:1 (v/v).

4.1.3 [Ru] (0.2 mM), MQ^+ (100 mM), phosphate (0.5 mM)

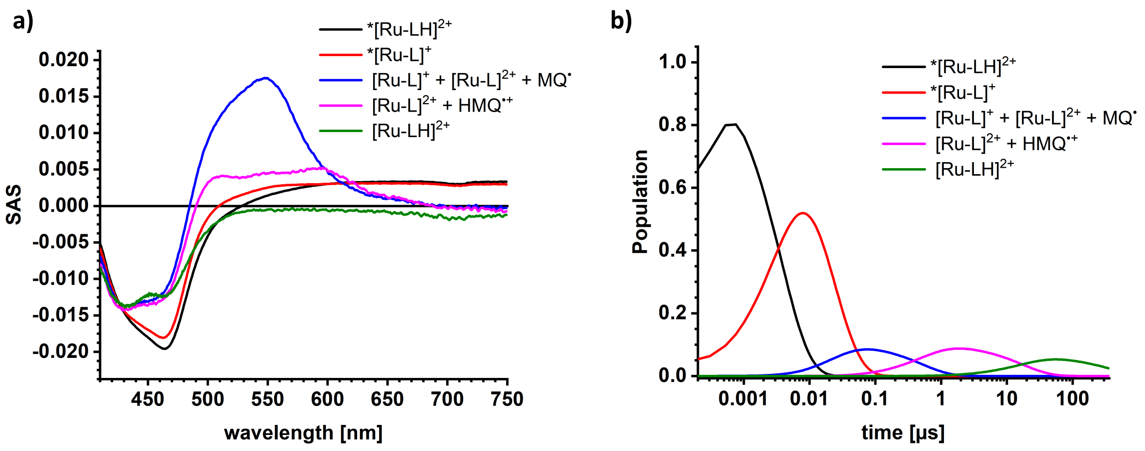

Supplementary Fig. 4.5. a, Species associated spectra (SAS) from target analysis of c(Ru) = 0.2 mM, c(MQ^+) = 100 mM and c(phosphate) = 0.5 mM in acetonitrile/water 1:1 (v/v) [2]. **b,** Corresponding time-population profiles of the different SAS.

4.1.4 [Ru] (0.2 mM), MQ^+ (100 mM), phosphate (5 mM)

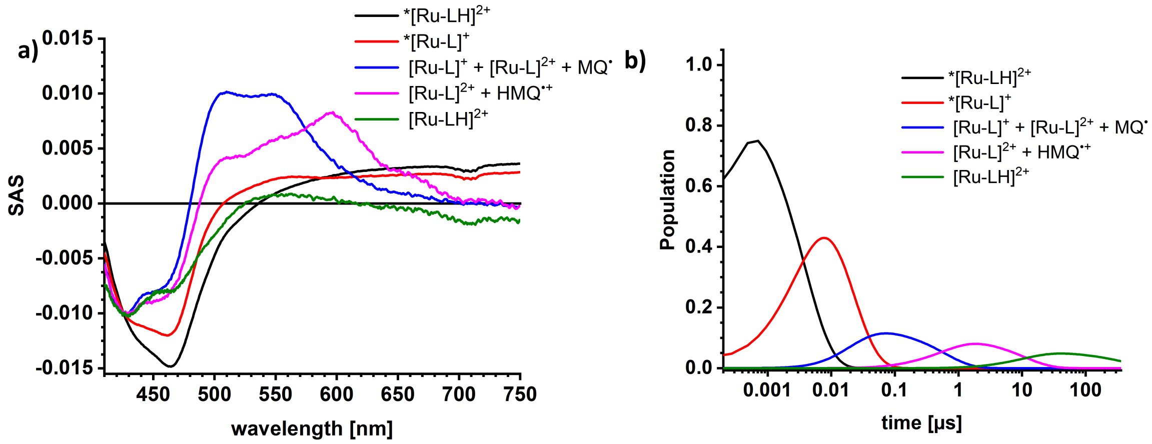

Supplementary Fig. 4.6. a, Species associated spectra (SAS) from target analysis of c(Ru) = 0.2 mM, c(MQ^+) = 100 mM and c(phosphate) = 5 mM in acetonitrile/water 1:1 (v/v) [2]. **b,** Corresponding time-population profiles of the different SAS.

4.1.5 Protonation of MQ• and Thermal Reverse PCET

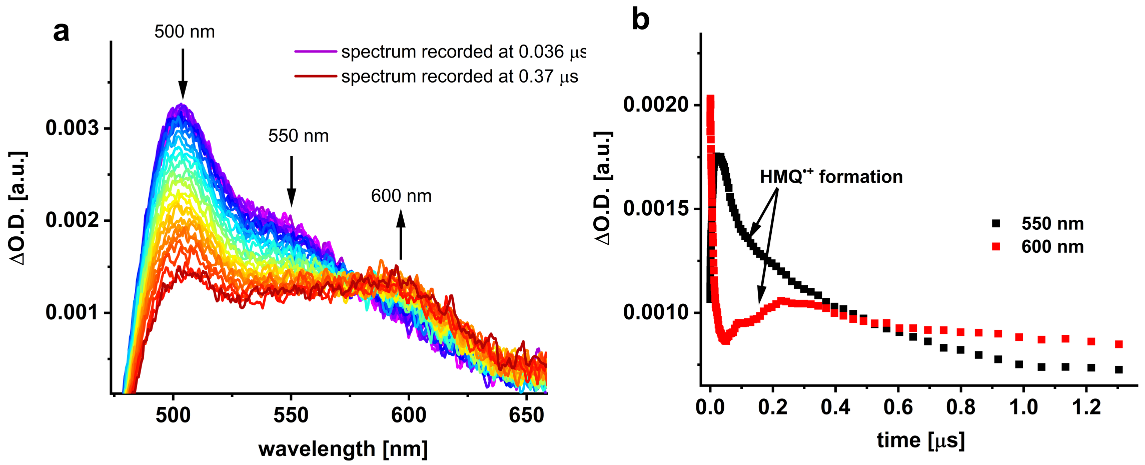

Supplementary Fig. 4.7. a, Spectral changes recorded within 0.036 – 0.37 µs after pulsed laser photoexcitation at 387 nm of 0.2 mM $[Ru(bpy)_2pyimH]^{2+}$ in the presence of 100 mM $[MQ^+]$ in water/acetonitrile solution buffered to a pH of 6.7 (50 mM phosphate buffer) at 25°C and ambient pressure. **b**, Kinetic traces recorded at 550 (black) and 600 nm (red) for the $MQ^{•+}$ protonation involving 50 mM phosphate buffer as proton donor after pulsed laser photoexcitation at 387 nm of 0.2 mM $[Ru(bpy)_2pyimH]^{2+}$ in the presence of 100 mM $[MQ^+]$ in water/acetonitrile solution buffered to a pH of 6.7 at 25°C and ambient pressure.

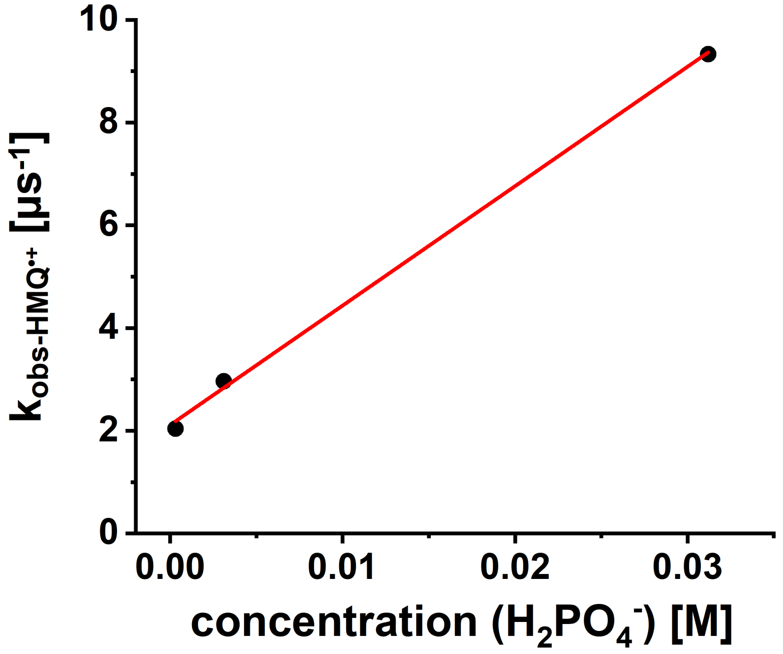

Supplementary Fig. 4.8. Dependence of pseudo-first order rate constants (µs^{-1}) for the MQ• protonation, $k_{obs\text{-}HMQ•+}$, on the phosphate buffer concentration. The parameters for the linear fit are summarized in Supplementary Table 3.1.

Note: $MQ^•$ is not a final reaction product. In the subsequent reaction step, the bands at 500 nm and 550 nm, associated with the products of intramolecular quenching and PCET-quenching pathways, respectively, decrease, while a new band at approximately 600 nm emerges (Supplementary Fig. 4.7, also see Fig. 5b,c,e,f). This new band arises from the protonation of **$MQ^•$**, leading to the formation of **$HMQ^{•+}$** (PT_2 in Fig. 1). The rate of **$HMQ^{•+}$** formation was investigated at three different phosphate buffer concentrations. However, it is worth noting that under such conditions, the varying concentration of the buffer not only affects the rate of **$MQ^•$** protonation but also influences the amount of **$MQ^•$** formed due to competition between intramolecular quenching to form **$[Ru\text{-}L]^+$** (path k_2 in Fig. 1) and the PCET-quenching to **$[Ru\text{-}L]^{2+}$ / $MQ^•$** (see Supplementary Fig. 4.3). Thus, the concentration of *in situ* generated **$MQ^•$** could not be maintained constant and therefore the effects of pressure on this reaction step were not further analyzed. Nevertheless, under ambient pressure, the second-order rate constant for the protonation of **$MQ^•$** by $H_2PO_4^-$ (PT_2 in Fig. 1, or k_5 in Extended Data Figure 1) was estimated to be $k_{HMQ•+} = (2.3 \pm 0.1) \times 10^8$ M^{-1} s^{-1}. We determined this using the slope of the linear dependence of pseudo-first order rate constants (μs^{-1}), $k_{obs\text{-}HMQ•+}$, on the phosphate buffer concentration (Supplementary Fig. 4.8). The significant intercept of $k_{p\text{-}HMQ•+} = 2.1 \pm 0.14$ μs^{-1} is ascribed to a parallel reaction, in which **$MQ^•$** undergoes protonation by water as a proton donor. Kinetics of a final step, which involves the thermal reverse PCET between **$[Ru\text{-}L]^{2+}$** and **$HMQ^{•+}$** to re-form the starting ground-state **$[Ru\text{-}LH]^{2+}$** and **MQ^+**, is depicted in Supplementary Fig. 4.9.

General remark: It is worth noting that the varying concentration of the buffer not only affects the rate of **$MQ^•$** protonation but also influences the amount of **$MQ^•$** formed due to competition between intramolecular quenching to form **$[Ru\text{-}L]^+$** (path k_2 in Fig. 1 in the main text) and the PCET-quenching to **$[Ru\text{-}L]^{2+}$** / **$MQ^•$** (see Supplementary Fig. 4.3). Thus, the concentration of *in situ* generated **$MQ^•$** could not be maintained constant and therefore the effects of pressure on this reaction step were not further analyzed.

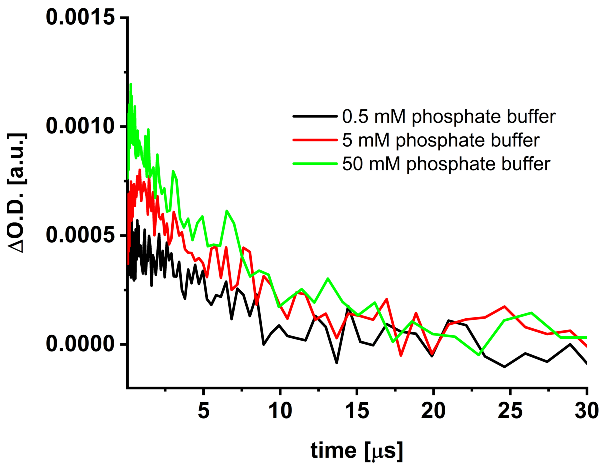

Supplementary Fig. 4.9. Kinetic traces recorded at 600 nm for the thermal reverse PCET between $HMQ^{\bullet+}$ and **Ru^{3+}-L^{-}** at different phosphate buffer concentrations (0.5 – 50 mM) after pulsed laser photoexcitation at 387 nm of 0.2 mM $[Ru(bpy)_2pyimH]^{2+}$ in the presence of 100 mM $[MQ^{+}]$ in water/acetonitrile solution buffered to a pH of 6.7 at 25°C and ambient pressure.

Note: A look at the kinetic traces associated with the $HMQ^{\bullet+}$ decay at 600 nm at various concentrations of the phosphate buffer (Supplementary Fig. 4.9) prompts to faster kinetics. Accelerating the reverse PCET as the buffer concentration is increased suggests that the buffer functions as a proton mediator. This is in fact similar to the PCET found for the forward reaction between $*[Ru\text{-}LH]^{2+}$ and MQ^{+}.

4.2 PIPES Buffered System

General remark: Due to the lower quality of the data when using PIPES buffer, the corresponding high-pressure data were not analyzed in details. Nevertheless, in PIPES buffer solutions with 100 mM **MQ^+**, ***[Ru-L]$^+$** is still discernable, which confirms, on one hand, no overall changes in a PCET mechanism upon variation of **MQ^+** concentrations and, on the other hand, the operation of stepwise ET/PT. Overall, our experimental observations were sufficient for mechanistic assignments. Notable are, however, some side-reactions, as the ground-state **[Ru-LH]$^{2+}$** bleach does not quantitatively recover. Factors that might affect a different mechanistic behavior at high **MQ^+** concentrations using PIPES buffer as a proton source will be addressed in future studies.

4.2.1 [Ru] (0.2 mM), MQ^+ (100 mM), PIPES (50 mM)

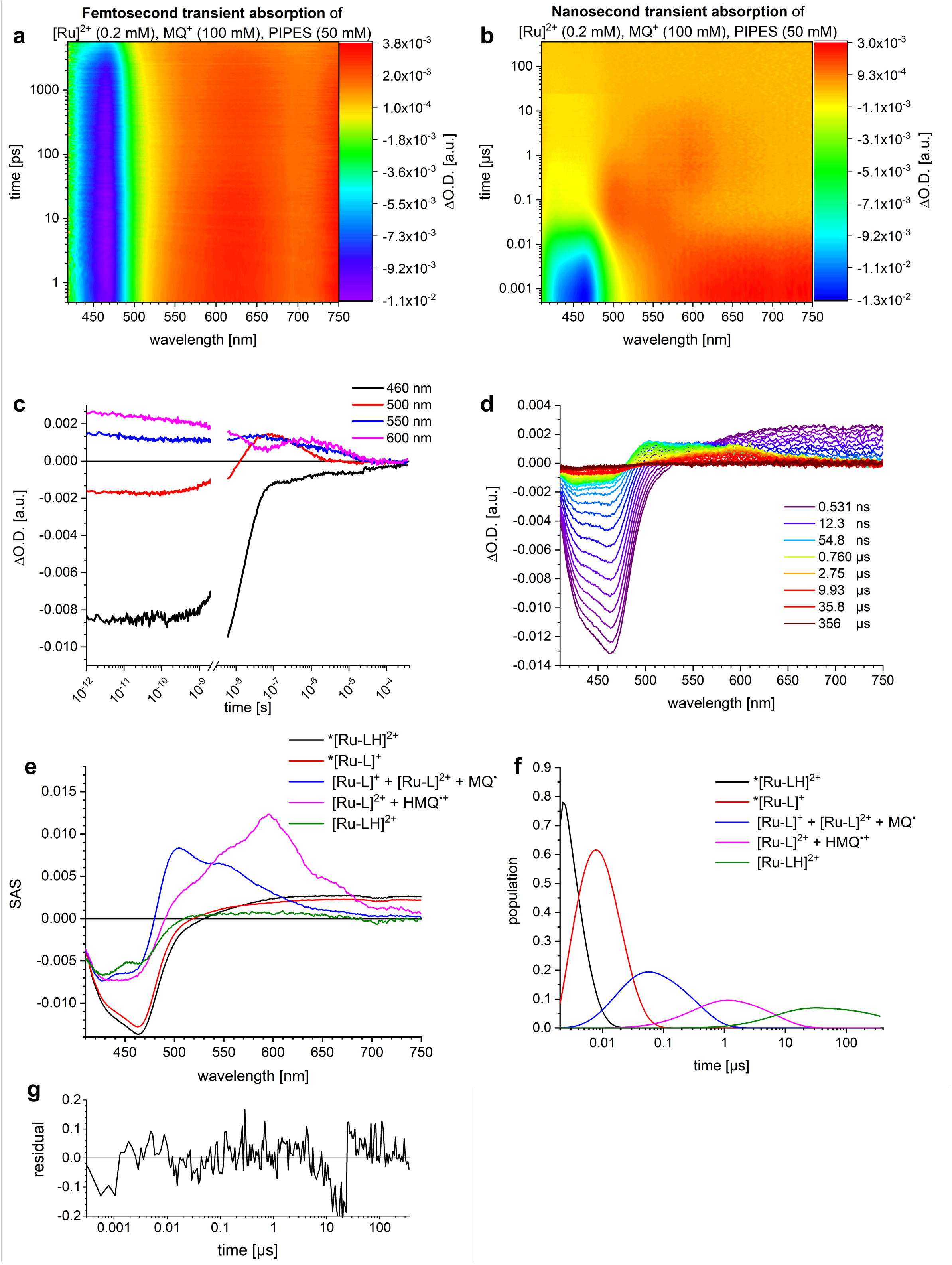

Supplementary Fig. 4.10. a, Zero point and chirp corrected fs-TAS spectra. **b,** Zero point and chirp corrected ns-TAS spectra. **c,** Time traces from fs- and ns-TAS spectra at 460 (black), 500 (red), 550 (blue), and 600 nm (purple). **d,** Spectral slices from the ns-TAS spectra. **e,** Species associated spectra (SAS) from target analysis of the ns-TAS spectra. **f,** Time evolution of the species associated spectra (SAS) from target analysis of the ns-TAS spectra [2]. **g,** Residual of the ns-TA spectra after fitting with target analysis. The jump in the residual traces at 80 μs shown in **g** is a result of an instrument artefact caused by light scattering.

The time traces on the (1 ps-1 ns) time scale and on the (1 ns-350 µs) time scale were measured with different spectrometers, hence the discrepancy of the ΔO.D. values. All spectra were recorded at room temperature in acetonitrile/water 1:1 (v/v) with the following composition: c([Ru(bpy)$_2$pyimH]$^{2+}$) = 0.2 mM, c(MQ$^+$) = 100 mM, c(PIPES) = 50 mM.

4.2.2 [Ru] (0.2 mM), MQ$^+$ (100 mM), PIPES (5 mM)

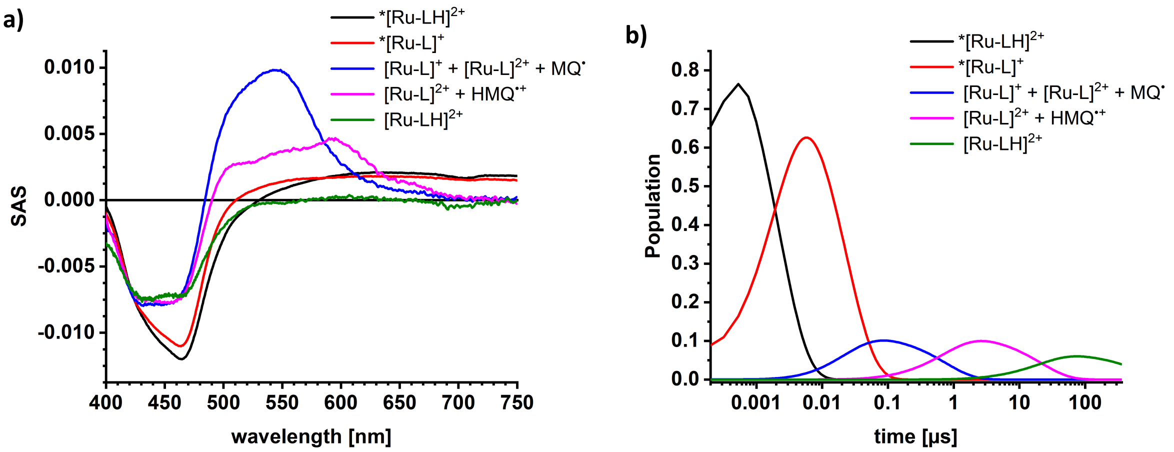

Supplementary Fig. 4.11. a, Species associated spectra (SAS) from target analysis of c(Ru) = 0.2 mM, c(MQ$^+$) = 100 mM and c(PIPES) = 5 mM in acetonitrile/water 1:1 (v/v) [2]. **b,** Corresponding time-population profiles of the different SAS.

4.2.3 [Ru] (0.2 mM), MQ$^+$ (100 mM), PIPES (0.5 mM)

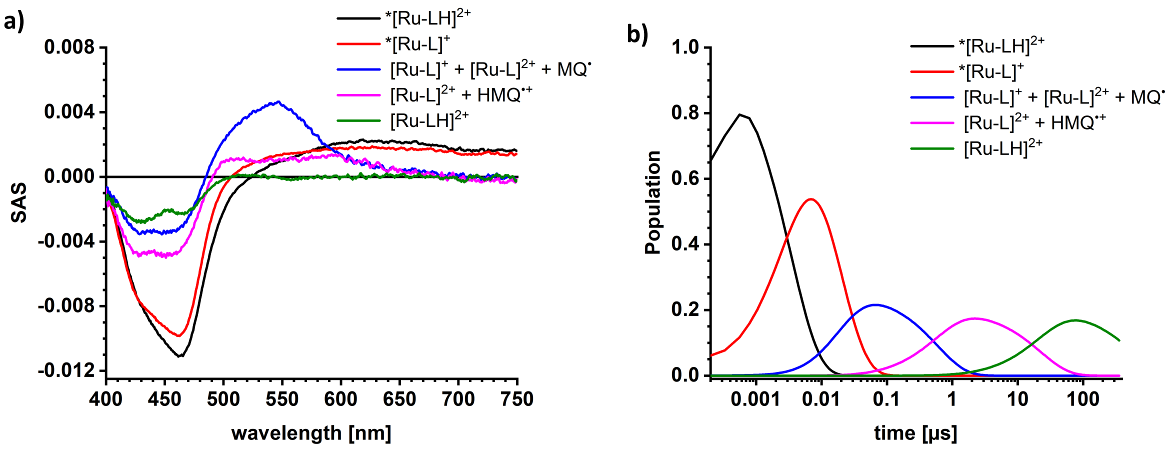

Supplementary Fig. 4.12. a, Species associated spectra (SAS) from target analysis of c(Ru) = 0.2 mM, c(MQ$^+$) = 100 mM and c(PIPES) = 0.5 mM in acetonitrile/water 1:1 (v/v) [2]. **b,** Corresponding time-population profiles of the different SAS.

4.3 Target Analysis in the presence of various MQ^+ concentrations at 50 mM Pipes Buffer

4.3.1 [Ru] (0.2 mM), MQ^+ (10 mM), PIPES (50 mM)

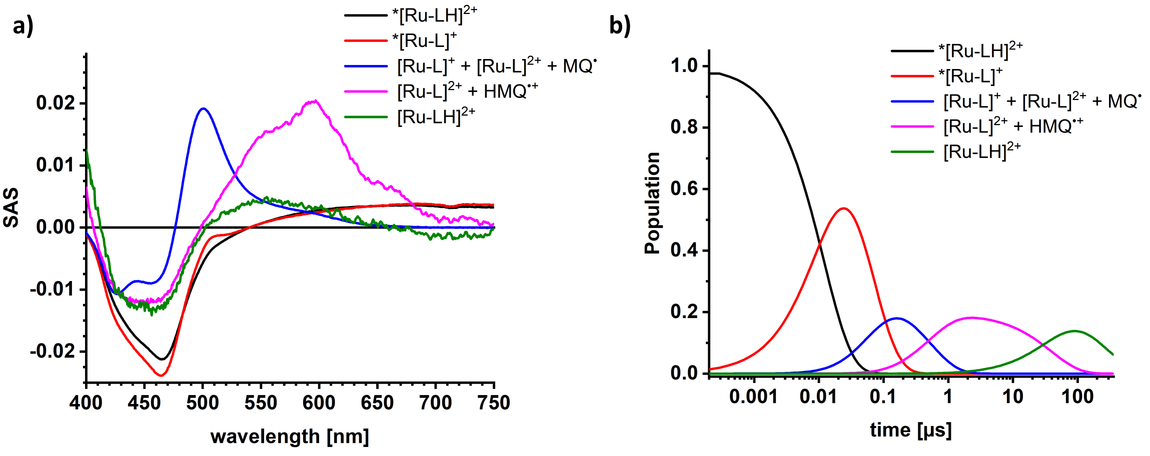

Supplementary Fig. 4.13. a, Species associated spectra (SAS) from target analysis of c(Ru) = 0.2 mM, c(MQ^+) = 10 mM and c(PIPES) = 50 mM in acetonitrile/water 1:1 (v/v) [2]. **b,** Corresponding time-population profiles of the different SAS.

4.3.2 [Ru] (0.2 mM), MQ^+ (1 mM), PIPES (50 mM)

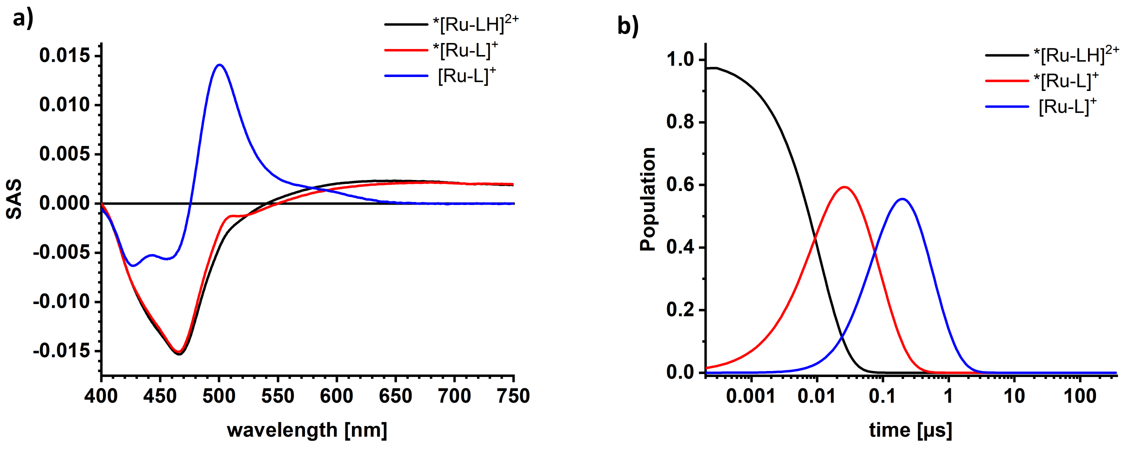

Supplementary Fig. 4.14. a, Species associated spectra (SAS) from target analysis of c(Ru) = 0.2 mM, c(MQ^+) = 1 mM and c(PIPES) = 50 mM in acetonitrile/water 1:1 (v/v). **b**, Corresponding time-population profiles of the different SAS.

5 References

(1) Pannwitz, A.; Wenger, O. S. Proton coupled electron transfer from the excited state of a ruthenium(II) pyridylimidazole complex. *Phys. Chem. Chem. Phys*. **2016**, 18 (16), 11374-11382.

(2) In the last SAS, notable are, however, some side reactions, as the ground state (**[Ru-LH]**$^{2+}$) bleach does not quantitatively recover.
